# Proteomic Identification of *Coxiella burnetii* Effector Proteins Targeted to the Host Cell Mitochondria During Infection

**DOI:** 10.1074/mcp.RA120.002370

**Published:** 2020-12-03

**Authors:** Laura F. Fielden, Nichollas E. Scott, Catherine S. Palmer, Chen Ai Khoo, Hayley J. Newton, Diana Stojanovski

**Affiliations:** 1 Department of Biochemistry and Molecular Biology and Bio21 Molecular Science and Biotechnology Institute, The University of Melbourne, Melbourne, Victoria, Australia; 2 Department of Microbiology and Immunology, University of Melbourne at the Peter Doherty Institute for Infection and Immunity, Melbourne, Victoria, Australia

**Keywords:** Host-pathogen interactions, Intracellular bacteria, T4SS, Bacterial effector protein, Coxiella burnetii, Mitochondria, protein targeting, Label-free quantitative proteomics, Organelle purification, Proteomics screen, AGC, automatic gain control, IMS, intermembrane space, IMM, inner mitochondrial membrane, LFQ, label-free quantitation, Mce, Mitochondrial *Coxiella* effector protein, PK, Proteinase K, PMSF, phenylmethylsulfonyl fluoride, T4SS, Type IV Secretion System, TCA, trichloroacetic acid, TIM, translocase of the inner mitochondrial membrane, THP-1 cells, human monocyte derived macrophage cell line, TOM, translocase of the outer mitochondrial membrane

## Abstract

Modulation of the host cell is integral to the survival and replication of microbial pathogens. Several intracellular bacterial pathogens deliver bacterial proteins, termed “effector proteins” into the host cell during infection by sophisticated protein translocation systems, which manipulate cellular processes and functions. The functional contribution of individual effectors is poorly characterized, particularly in intracellular bacterial pathogens with large effector protein repertoires. Technical caveats have limited the capacity to study these proteins during a native infection, with many effector proteins having only been demonstrated to be translocated during over-expression of tagged versions. Here, we developed a novel strategy to examine effector proteins in the context of infection. We coupled a broad, unbiased proteomics-based screen with organelle purification to study the host–pathogen interactions occurring between the host cell mitochondrion and the Gram-negative, Q fever pathogen *Coxiella burnetii*. We identify four novel mitochondrially-targeted *C. burnetii* effector proteins, renamed Mitochondrial *Coxiella* effector protein (Mce) B to E. Examination of the subcellular localization of ectopically expressed proteins confirmed their mitochondrial localization, demonstrating the robustness of our approach. Subsequent biochemical analysis and affinity enrichment proteomics of one of these effector proteins, MceC, revealed the protein localizes to the inner membrane and can interact with components of the mitochondrial quality control machinery. Our study adapts high-sensitivity proteomics to study intracellular host–pathogen interactions, providing a robust strategy to examine the subcellular localization of effector proteins during native infection. This approach could be applied to a range of pathogens and host cell compartments to provide a rich map of effector dynamics throughout infection.

## Highlights

• Mitochondrial purification and proteomics to study host–pathogen interactions.
• Quantitative proteomics reveals *Coxiella* effector proteins at mitochondria.
• Insights into effector protein targeting during native infection.
• Further characterization of MceC reveals localization and interaction.

## Graphical Abstract

**Figure:**
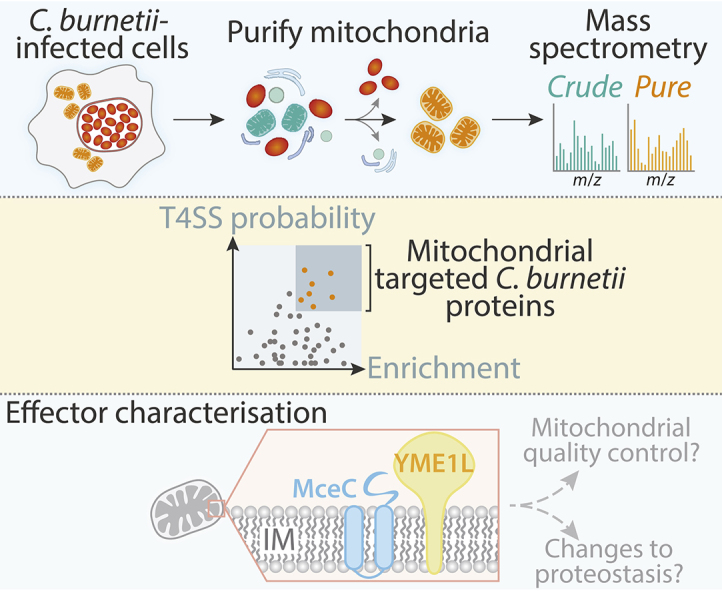

## In Brief

A broad, unbiased proteomics-based screen with organelle purification to study the host–pathogen interactions occurring between the host cell mitochondrion and the Q fever pathogen *Coxiella burnetii*. This reveals a subset of *Coxiella* effector proteins at mitochondria during infection. Our study adapts high-sensitivity proteomics, providing a robust strategy to examine the subcellular localization of effector proteins during native infection.

Numerous microbial pathogens have evolved strategies to survive within the host cell. During infection, a subset of bacterial virulence factors, termed effector proteins, are delivered into the host cell where they function to modulate host cell processes and ultimately contribute to bacterial pathogenesis. Bacterial effector proteins are translocated into the host cell by specialized secretion systems (1, 2). The repertoire of effector proteins encoded by a bacterial pathogen is unique, reflective of the intracellular niche it occupies and varies greatly across different bacterial species (3, 4). Bacteria possessing a Dot/Icm type IV secretion system (hereafter referred to as T4SS) typically encode a large effector cohort, for instance the respiratory pathogen *Legionella pneumophila* encodes for approximately 330 effector proteins and the evolutionarily related, *Coxiella burnetii* for approximately 150 (5, 6). The substantial number of proteins potentially delivered by these bacterial pathogens into the host cell creates a problem in identifying *bona fide* translocated effectors and delineating the function of these proteins during infection.

Approaches to biochemically characterizing effector proteins typically concentrate on one effector in isolation, often removed from the context of infection. The low abundance of effector proteins within the host cell creates a technical challenge that has limited study during native infection. Often, over-expression or “tagging” of effectors with a protein or peptide label is employed to enable localization and interaction studies. However, our increasing understanding of the manner in which bacterial secretion systems deliver protein substrates into the host cell and the important role of meta-effector interactions during infection highlights the risks associated with an individualistic approach (7, 8, 9, 10, 11). The rapidly expanding field of high-sensitivity mass spectrometry coupled to subcellular organelle isolation presents a solution to the study of endogenous host–pathogen interactions occurring during infection, particularly for bacterial pathogens harboring large effector repertoires, where functional redundancy exists within the effector cohort.

*C. burnetii* is a Gram-negative, obligate intracellular bacterial pathogen and the causative agent of the zoonotic disease Query (Q) fever in humans (12). Q fever is a complex disease with a range of clinical presentations from asymptomatic seroconversion to acute illness and debilitating chronic infection (13, 14, 15, 16). Human infection occurs by inhalation of contaminated aerosols and the bacterium preferentially infects alveolar macrophages (17). *C. burnetii* encodes a T4SS which is essential for intracellular replication of the bacterium (18, 19). This translocation system mediates the delivery of bacterial effector proteins into the host cell over the course of infection (20, 21). Thus far, 150 proteins have been identified with the capacity for T4SS translocation; however, whether these are all delivered into the host cell during native infection is unknown. The identification and characterization of substrates of the *C. burnetii* T4SS has been a rapidly expanding area of research, greatly assisted by the development of axenic culture and genetic manipulation techniques (18, 22, 23, 24, 25, 26, 27). Despite significant advancement in our identification of these bacterial virulence factors, the majority of *C. burnetii* effector proteins remain functionally uncharacterized.

We previously demonstrated that the *C. burnetii* effector protein MceA specifically targets the host cell mitochondria during infection (28). This research highlighted the organelle as a *bona fide* target of *C. burnetii* during infection. Mitochondria are essential eukaryotic organelles, fundamental to cell function and survival. Mitochondria contribute to metabolism and bioenergetics, iron-sulfur cluster biogenesis, lipid synthesis, calcium homeostasis, immune signaling, and apoptosis (29, 30, 31). Given this crucial role in cell biology and the important involvement of mitochondria in the host response to infection, mitochondrial functions are frequently targeted by microbial pathogens during infection of the host cell (32, 33, 34). For instance, *L. pneumophila* targets mitochondrial dynamics and metabolism via the T4SS effector protein, mitochondrial fragmentation factor (35). Mitochondrial fragmentation factor targets host cell Ran GTPase promoting DRP1-dependent fragmentation of the host mitochondria and assisting in biphasic regulation of mitochondrial respiration in a macrophage host (35).

Currently, little is known about the interaction between *C. burnetii* and the host cell mitochondrion during infection. Earlier studies demonstrated that the bacterium has the ability to block apoptosis by preventing the release of cytochrome *c* from the mitochondria (36). The ability to inhibit the mitochondrial pathway of apoptosis has highlighted two additional *C. burnetii* effector proteins, AnkG and CaeB, that appear to target the organelle; however, characterization of these effectors was performed in the absence of infection (37, 38). In consideration of the large cohort of bacterial proteins delivered to the host cell by *C. burnetii* and the essentiality of multiple mitochondrial functions to cellular homeostasis, we hypothesized that additional effector proteins would localize to the organelle during infection. Here, an unbiased, unlabeled proteomics approach was utilized to identify *C. burnetii* effector proteins associated with the host cell mitochondria during infection. This approach established a list of seven candidate *C. burnetii* proteins targeted to the host cell mitochondria and validated the association of four proteins with the organelle which we renamed MceB–E. We further characterize one of these candidates, CBU1425 (renamed MceC), for confirmation of submitochondrial localization and protein interactions. MceC was found to be imported into the organelle and integrated into the inner membrane. Furthermore, we demonstrate that MceC forms associations with members of a mitochondrial inner membrane quality control network. Development and use of this unbiased proteomics-based technique has allowed us to uncover host–pathogen interactions between *C. burnetii* and the mitochondria in the context of native infection and provides an exciting platform for future exploration of effector interactions with host cell organelles.

## Experimental Procedures

### Bacterial Strains and Culturing Conditions

Plaque purified *C. burnetii* Nine Mile Phase II (NMII), strain RSA439 clone 4 wildtype and wildtype expressing mCherry were used in this study. *C. burnetii* strains were cultured axenically in liquid Acidified Citrate Cysteine Medium 2 (ACCM-2) with chloramphenicol (3 μg/ml, Boehringer Mannheim) when required for 7 days at 37 °C, 5% CO_2_, and 2.5% O_2_, as previously described (22, 39). *Escherichia coli* XL1-Blue used for plasmid construction and propagation were cultured in LB broth or agar plates containing ampicillin (50 μg/ml, Sigma) as appropriate.

### Mammalian Cell Lines and Culturing, Transient Transfection, and Stable Cell Line Generation

Cell lines used in this study were THP-1 (human monocytic leukemia) cells, HeLa (Henrietta Lacks, human cervical carcinoma cells, CCL2), and HEK293T Flp-In T-REx 293 (Thermo Fisher Scientific). THP-1 cells were cultured in Roswell Park Memorial Institute 1640 medium containing 10% heat-inactivated fetal bovine serum (Thermo Fisher Scientific). HeLa and HEK293 cells were cultured in Dulbecco's Modified Eagle Medium (Gibco) containing 5 to 10% fetal bovine serum (Thermo Fisher Scientific). All cells were maintained at 37 °C, 5% CO_2_.

Transient transfections were performed using Lipofectamine 3000 (Thermo Fisher Scientific) transfection reagent as per the manufacturer's instructions. Stable tetracycline-inducible cell lines were generated in accordance with the manufacturers protocol and as previously described (40). Cells at a confluency of ∼60% were co-transfected with pcDNA5-FRT/TO-CBU1425-3XFLAG and pOG44 (encoding the Flp-recombinase) at a 1:9 ratio (per ng of DNA). Three days post-transfection, positive clones were selected for using Hygromycin B (200 μg/ml) and applied until single colonies could be detected and foci resuspended and transferred for recovery and expansion. Protein expression was induced by the addition of tetracycline (1 μg/ml) for desired time.

### *C. burnetii* Quantitation and Infections

THP-1 cells were seeded at a density of 1.5 × 10^7^ cells per well into 15 cm tissue culture plates and treated with 10 nM phorbol 12-myristate 13-acetate for 3 days to induce differentiation into a macrophage-like cell. *C. burnetii* quantitation was performed using quantitative PCR with gene specific primers for *ompA* (forward: 5′-CAGAGCCGGGAGTCAAGCT-3′, reverse: 5′-CTGAGTAGGAGATTTGAATCGC-3′) to provide a multiplicity of infection of 100 (41). Cells were incubated with bacteria at 37 °C, 5% CO_2_ for 24 h, washed once in warm 1× PBS, media replaced with fresh Roswell Park Memorial Institute 1640 with 10% fetal bovine serum and incubated for a further 48 h before collection for mitochondrial isolation and proteomics analysis.

### Immunofluorescence Microscopy

Cells were fixed in paraformaldehyde (4% (w/v) in PBS containing 5% (w/v) sucrose) for 10 to 15 min and then permeabilized in 0.1% (v/v) TX-100 in PBS at RT. Coverslips were blocked in 3% (w/v) BSA in PBS for 30 min at RT, followed by incubation with primary and secondary antibodies diluted in 3% (w/v) BSA in PBS. Coverslips were incubated with primary antibodies for 1 to 2 h at RT, washed with PBS (three changes over 10 min) followed by incubation with secondary antibodies for 30 to 60 min (1:500 dilution; goat anti-mouse AlexaFluor 488 conjugate [Invitrogen] or goat anti-rabbit AlexaFluor 568 conjugate [Invitrogen]). Coverslips were washed with PBS containing Hoechst stain (10 μg/ml; Invitrogen), affixed onto glass slides using mounting media (0.2 M DABCO [Sigma], 0.1 M Tris-HCl (pH 8.0), 90% glycerol). Cells were imaged on a Leica SP8 confocal microscope and image analysis performed using ImageJ/FIJI software (https://imagej.nih.gov/ij/) (42, 43). For analysis of fluorescence profile, the fluorescence profile of regions of interest were obtained using the Fiji plugin Dynamic ROI Profiler. Fluorescence intensity measurements of 3 μm regions were recorded and graphed in Microsoft Excel for green and red fluorescence (arbitrary units).

### Mitochondrial Isolation and Purification

Mitochondria were isolated by differential centrifugation (44). Isolated cells were resuspended in solution A (70 mM sucrose, 220 mM mannitol, 20 mM HEPES-KOH (pH 7.6), 1 mM EDTA, 0.1 mM phenylmethylsulfonyl fluoride [PMSF] and 2 mg/ml BSA) and homogenized in a handheld glass Dounce homogenizer (typically 15–20 strokes). The homogenate was centrifuged at 600*g* for 5 min at 4 °C to remove nuclear and cellular debris. The supernatant was centrifuged at 12,000*g* for 10 min at 4 °C following which the pellet (crude mitochondrial fraction) was resuspended in solution B (70 mM sucrose, 220 mM mannitol, 20 mM HEPES-KOH [pH 7.6] and 1 mM EDTA). Mitochondria isolated from *C. burnetii*-infected cells were further purified using sucrose density gradient centrifugation (45) and affinity purification. Following crude isolation, mitochondria were resuspended in continuous gradient buffer (0.25 M sucrose, 10 mM Tris-Cl [pH 7.4] and 1 mM EDTA) and overlaid on a 34 to 64% continuous sucrose gradient with a 67% sucrose cushion at the base of the tube (34/64/67% [w/v] sucrose, 10 mM Tris-Cl [pH 7.4] and 1 mM EDTA). Gradients were centrifuged at 170,000*g* for 1 h in a SW41Ti swinging bucket rotor (Beckman Coulter) at 4 °C. Following ultracentrifugation, 12 × 1 ml fractions were removed, and fractions 2 to 5 were pooled, diluted 1:2 with continuous gradient buffer and centrifuged at 16,000*g* for 10 min at 4 °C to isolate mitochondria. Mitochondria were further purified by affinity purification using anti-TOM22 microbeads (Miltenyi Biotec). Mitochondria were resuspended in 1 ml solution B containing protease inhibitor (PI; Roche) and incubated with anti-TOM22 microbeads on a rotary wheel for 30 min at 4 °C. The mitochondrial resuspension was transferred to a MACS LS column (Miltenyi Biotech), and bound mitochondria were washed 3× with solution B containing PI and eluted in solution B containing PI. Purified mitochondria were re-isolated and prepared for analysis by mass spectrometry. Mitochondrial protein concentration was estimated using UV spectrophotometry or BCA protein assay (Pierce, Thermo Fisher Scientific).

### Gel Electrophoresis and Immunoblot Analysis

Tris-tricine SDS-PAGE was performed as previously described (46, 47, 48, 49). Samples for electrophoresis were combined with SDS-PAGE loading dye (50 mM Tris-Cl [pH 8.45], 100 mM dithiothreitol, 2% [w/v] SDS, 10% [v/v] glycerol, 0.1% [w/v] bromophenol blue). Electrophoresis was performed in the presence of Tris-tricine SDS-PAGE cathode buffer (0.1 M Tris, 0.1 M Tricine [pH 8.45], 0.1% [w/v] SDS) and anode buffer (0.2 M Tris-Cl [pH 8.9]).

Western transfers were performed using the Owl HEP-1 Semidry Electroblotting System (ThermoFisher Scientific) semi-dry transfer method. Following electrophoresis, SDS-PAGE gels were transferred onto a polyvinylidene fluoride membrane (Millipore). Polyvinylidene fluoride membranes (whole or in strips) were subjected to immunoblot analysis with specific primary antibodies and secondary antibodies (anti-mouse/rabbit IgG coupled to horseradish peroxidase; Sigma Aldrich; 1:5000 dilution in blocking buffer). Detection of chemi-luminescent signal was performed with Clarity ECL Western Blotting substrate (BioRad) and imaged using a ChemiDoc MP Imaging system (BioRad). Immunoblot quantitation was performed on three independent biological replicates using Image Lab software (BioRad).

### Whole Cell Lysate Preparation

Isolated cells were washed in ice-cold PBS and cell pellets resuspended in RIPA buffer for cell lysis (50 mM Tris-Cl [pH 8.0], 150 mM NaCl, 1% [v/v] TX-100, 0.5% [w/v] sodium deoxycholate, 0.1% [w/v] SDS). Supernatants were collected, and protein amount quantitated using BCA protein assay kit (Pierce, Thermo Fisher Scientific). 80 to 100 μg protein was loaded per lane for SDS PAGE.

### Mitochondrial Treatments

For mitochondrial subfractionation, samples (100 μg) were resuspended in (i) solution B, (ii) swelling buffer (10 mM HEPES-KOH, pH 7.4), or (iii) solubilization buffer (1% [v/v] TX-100) and incubated on ice for 5 min. Samples were incubated for a further 10 min in either the absence or the presence of PK (50 μg/ml) followed by addition of 1 mM PMSF for 5 to 10 min. All samples were trichloroacetic acid (TCA) precipitated and analyzed by SDS-PAGE and immunoblotting. For sodium carbonate extraction, isolated mitochondria (200 μg) were resuspended in 100 mM Na_2_CO_3_ (pH 11) and incubated on ice for 30 min with occasional agitation. Samples were centrifuged at 100,000*g* for 30 min at 4 °C to separate integral membrane proteins (pellet fraction) and soluble proteins (supernatant fraction) and then TCA precipitated. For protease protection assays, ∼80 μg of mitochondrial protein was resuspended at 1 μg/μl in solubilization buffer (50 mM Tris-Cl [pH 7.4], 150 mM NaCl) containing 0 to 1% [v/v] digitonin and solubilized for 1 h at 4 °C. Samples were split and either left untreated or treated with PK (50 μg/ml) for 10 min followed by addition of PMSF (1 mM) for 10 min. Samples were TCA precipitated, separated by SDS-PAGE and analyzed by immunoblotting.

### Immunoprecipitation Analysis

Isolated crude mitochondrial samples (3.5–5 mg) were resuspended in digitonin solubilization buffer (1% [v/v] digitonin, 50 mM Tris-Cl [pH 7.4], 150 mM NaCl, 1x PI [Roche]) at 2 mg/ml and incubated, end-over-end at 4 °C for 30 min. Solubilized mitochondria were clarified by centrifugation at 16,000*g* for 10 min at 4 °C. The supernatant was diluted to a final detergent concentration of 0.1% digitonin and incubated with pre-equilibrated anti-FLAG resin (Sigma Aldrich- A2220; 5–10 μl of resin/mg mitochondrial protein), end-over-end for 30 min at 4 °C. Resin and bound proteins were transferred to a Pierce spin column (Thermo Fisher Scientific-69705) and washed 8× in solubilization buffer containing 0.1% [w/v] digitonin. Bound proteins were eluted in 0.2 M glycine (pH 2.5) over 2 by 5 min incubations and elution fractions pooled. Total, supernatant, unbound, wash, and elution fractions were TCA precipitated, and pellet fraction directly resuspended into SDS-PAGE loading dye. All fractions were analyzed by SDS-PAGE and immunoblotting or elution fraction prepared for analysis by mass spectrometry.

### Mass Spectrometry

Mitochondrial protein (50–100 μg) was acetone-precipitated (8 volumes acetone, 1 volume water, 1 volume sample) overnight at −20 °C. Precipitated material was centrifuged at 16,000*g* for 10 min at 4 °C and dried at 65 °C for 5 to 10 min. Pellets were resuspended in urea (6 M, Sigma), thiourea (2 M, Sigma), and DTT (10 mM, Austral Scientific) and incubated at RT in the dark for 1 h to reduce disulfide bonds. Samples were alkylated by the addition of chloroacetamide (40 mM) and incubated for 1 h. Alkylation was halted by the addition of DTT (50 mM) and incubation at RT for 15 min. Samples were digested with Lys-C (1/200 [w/w]; Wako Lab Chemicals) for 4 h at RT before dilution with ammonium bicarbonate (20 mM; Sigma) and digestion with trypsin (1/50 [w/w]; Sigma) overnight at 25 °C. Digested peptides were acidified by the addition of formic acid (2% [v/v]; Sigma) and desalted using in-house C18 stage tips (Empore C18, Sigma) and dried down for liquid chromatography mass spectrometry (LC-MS) (50, 51). Before loading, samples were reconstituted in MS running buffer (2% acetonitrile [ACN, Sigma], 0.1% trifluoroacetic acid [Sigma]) to a concentration of 0.5 μg/μl of which 2 μg was loaded for mass spectrometry.

LFQ mass spectrometry mitochondria of *C. burnetii*^mCherry^-infected THP-1 cells were analyzed by LC-MS on an Orbitrap Lumos mass spectrometer coupled to a Dionex Ultimate 3000 Ultra-Performance Liquid Chromatography using a two-column chromatography set up composed of a PepMap100 C18 20 mm × 75 μm trap and a PepMap C18 500 mm × 75 μm analytical column (Thermo Fisher Scientific). Samples were concentrated at 5 μl/min onto the trap column for 5 min before the trap column was switched in-line with the analytical column using Buffer A (0.1% formic acid, 5% DMSO). Analytical separation was performed at 300 nl/min using a nonlinear ACN gradient by altering the composition of Buffer B (0.1% [v/v] formic acid, 94.9% [v/v] ACN, 5% [v/v] DMSO) from 3% to 22% over 180 min then from 30% to 40% over 7 min, 40% to 90% over 5 min, holding at 90% for 5 min, then dropped to 3% Buffer B over 3 min with the column then equilibrated by holding at 3% Buffer B for 5 min. Data were collected in positive mode using Data Dependent Acquisition with a 120,000 orbitrap resolution MS1 scan of mass-to-charge (m/z) range of 375 to 1500 (automatic gain control [AGC] set to 4 × 10^5^ or a maximum injection time of 50 ms) acquired every 3 s followed by MS2 scans. MS2 scans were acquired using high-energy collision dissociation fragmentation with a normalized collision energy of 35%, with an isolation window of 1.6 in the quadrupole, resolution of 7500 and an AGC of 5 × 10^4^ or a maximum injection time of 22 ms. A dynamic exclusion of duration of 30 s was applied for repeated precursors. Raw files were analyzed using MaxQuant platform (version 1.6.2.10) and searched against Uniprot human database (Accession: UP000005640; 73,101 entries, downloaded October 2018) and *C. burnetii* database (Accession: UP000002671; 1812 entries, downloaded from Uniprot October 2017), containing reviewed, canonical, and isoform variants and a database containing common contaminants generated by the Andromeda search engine (52). LFQ search parameters were left as default with Trypsin/P specificity with a maximum of two missed cleavages. Searches were performed with cysteine carbamidomethylation as a fixed modification and methionine oxidation, N-terminal acetylation, and methionine oxidation as variable modifications. FDR was determined using the target-decoy approach set to 2% for peptides and 1% for proteins. Unique and razor peptides were used for identification with a minimum ratio count of 2. A search tolerance of 4.5 ppm was used for MS1 and 20 ppm for MS2 matching. “Re-quantify” and “match between runs” functions were enabled with a match time window of 0.7 min (53).

MceC^3XFLAG^ immunoprecipitation samples were analyzed by LC-MS on an Orbitrap Elite mass spectrometer coupled to a Dionex Ultimate 3000 Ultra-Performance Liquid Chromatography (ThermoFisher Scientific) using a two-column chromatography set up composed of a PepMap100 C18 20 mm × 75 μm trap and a PepMap C18 500 mm × 75 μm analytical column (Thermo Fisher Scientific). Samples were concentrated at 5 μl/min onto the trap column for 5 min. Analytical separation was performed at 300 nl/min using a 65 min nonlinear gradient by altering the concentration of Buffer B (5% [v/v] DMSO, 94.9% [v/v] can, and 0.1% [v/v] formic acid) from 0 to 3% over 5 min, 3% to 22% over 32 min, then from 22% to 40% over 10 min, 40 to 80% over 5 min and held at 80% for 5 min then dropped to 3% over 3 min with the column equilibrated by holding at 3% for 10 min. Data were collected in positive mode using Data Dependent Acquisition with a 100,000 orbitrap resolution MS1 scan of mass-to-charge (m/z) range of 300 to 1650. The top 20 most intense precursor ions were subjected to rapid collision induced dissociation with normalized collision energy of 30 and activation q of 0.25. A dynamic exclusion of duration of 30 s was applied for repeated precursors. Raw files were analyzed using MaxQuant platform (version 1.6.10.43) and searched against Uniprot human database (73,101 entries, downloaded October 2018) and fasta sequence for the ORF of CBU1425 (downloaded from Uniprot October 2019). LFQ search parameters were left as default with Trypsin/P specificity with a maximum of two missed cleavages. Oxidation of methionine and N-terminal acetylation were specified as variable modifications. Carbamidomethylation of cysteine was set as a fixed modification. A search tolerance of 4.5 ppm was used for MS1 and 0.5 Da for MS2 matching. FDR was determined using the default target-decoy approach set to 1% for both peptides and proteins. Match between runs was enabled with a match time window of 0.7 min.

Mitochondria isolated from HEK293 MceC^3XFLAG^ or empty vector (EV) cell lines during expression time course were analyzed on a Q Exactive Plus orbitrap mass spectrometer (ThermoFisher Scientific) coupled to a Dionex Ultimate 3000 Ultra-Performance Liquid Chromatography (ThermoFisher Scientific). Peptides were injected into the trap column (PepMap100 C18 20 mm × 100 μm) at a flow rate of 5 μl/min before changing the trap in-line with the analytical column (PepMap C18 500 mm × 75 μm). Analytical separation was performed at 300 nl/min nonlinear gradient by altering the composition of Buffer B from 3% to 22% over 105 min then from 30% to 40% over 7 min, 40% to 90% over 5 min, holding at 90% for 5 min then dropped to 3% Buffer B over 3 min with the column then equilibrated by holding at 3% Buffer B for 5 min over 130 min. Data were collected in positive mode using Data Dependent Acquisition with a 70,000 orbitrap resolution MS1 scan of mass-to-charge (m/z) range of 375 to 1400 (maximum injection time of 50 ms, AGC target of 3 × 10^6^). The top 15 most intense precursor ions were subjected to MS2 using high-energy collision dissociation fragmentation with a normalized collision energy of 28% (resolution of 17,500, AGC target of 5 × 10^4^ and maximum injection time of 50 ms). Raw files were analyzed using MaxQuant platform (version 1.6.5.0) and searched against Uniprot human database (73,101 entries, downloaded October 2018) and fasta sequence for the ORF of CBU1425 (downloaded from Uniprot October 2019). LFQ search parameters were left as default with semispecific Trypsin/P digest with zero missed cleavages to assess if any alterations in proteostasis could be observed. Oxidation of methionine and N-terminal acetylation were specified as variable modifications. Carbamidomethylation of cysteine was set as a fixed modification. A search tolerance of 4.5 ppm was used for MS1 and 20 ppm for MS2 matching. FDR was determined using the default target-decoy approach set to 1% for both peptides and proteins. Match between runs was enabled with a match time window of 0.7 min.

### Data Analysis in Perseus

The “protein groups” output file was imported into the Perseus platform for further processing (54). Identifications labeled by MaxQuant as “only identified by site”, “potential contaminant”, and “reverse hit” were removed, and LFQ values normalized by log_2_-transformation. Identifications were matched to human and *C. burnetii* annotation files (downloaded from Uniprot at the same time as databases) using gene name identifiers. Mitochondrial proteins were annotated using the human MitoCarta database (55, 56). Data were exported into Excel and then into the R framework (https://www.r-project.org) integrated with R studio (https://rstudio.com/products/rstudio/) for generation of graphics.

### Bioinformatic Analysis of *C. burnetii* Proteins Using S4TE

*C. burnetii* proteins were analyzed and scored based on probability of translocation via the T4SS using the online bioinformatics program Searching Algorithm for Type IV Effector proteins (S4TE) 2.0, (https://sate.cirad.fr) (57). Preloaded *C. burnetii* Nine Mile RSA493 chromosome and plasmid (pQpH1) genbank sequences were analyzed using default settings (https://sate.cirad.fr/S4TE.php). Data were exported to Microsoft Excel and unannotated effector proteins manually annotated. Data were imported into the R framework (https://www.r-project.org) integrated with R studio (https://rstudio.com/products/rstudio/) for comparison with *C. burnetii* protein enrichment alongside purified mitochondria and generation of graphics.

### Experimental Design and Statistical Rationale

For label-free quantitative LC-MS/MS experiments of mitochondria, statistical analysis of data was consistent with published analyses from our and other groups employing similar instrumentation and methods (48, 58, 59). All experiments were performed in at least 3 independent biological replicates. Data were imported into Perseus analysis platform and LFQ values log2 transformed. Enrichment ratio was calculated on normalized data, requiring a minimum of 2 valid values in either “crude” or “pure” mitochondria group. Imputation (downshifted by 1.8σ with a distribution of 0.3σ) was applied to missing values in both groups population missing datapoints with values equivalent to the limit of detection in each experiment. Individual replicate enrichment ratios were averaged and compared with S4TE T4SS translocation probability scores. Proteins with an enrichment ratio greater than 0.85 and translocation score greater than 72 were considered highly enriched at the mitochondria. MceC affinity enrichment analysis were performed on mitochondria isolated from three biological replicates for each control and MceC-expressing cells requiring a minimum of three valid values in replicates from MceC-expressed condition. The fold change value used for significance in MceC affinity enrichment analysis was determined through a two-sided t-test based with multiple hypothesis undertaken using a permutation-based FDR with an FDR of 5% and s0 value of 1. Imputation (downshifted by 1.8σ with a distribution of 0.3σ) was applied to missing values. Label-free MceC time course experiments were performed on three biological replicates per time point. Imputation (downshifted by 1.8σ with a distribution of 0.3σ) was applied to missing values in both groups population missing datapoints with values equivalent to the limit of detection in each experiment. An ANOVA test with multiple hypothesis using a permutation-based FDR with an FDR of 5% and an s0 value of 0 was used to identify proteins significantly altered across all time points.

### Antibodies

Antibodies used in this study include anti-FLAG monoclonal (F1804; Sigma); anti-FLAG polyclonal (PA1-984B; Thermo Fisher Scientific); mCherry (NBP2-25157; Novus Biologicals); β-actin (A5316; Sigma); Cytochrome *c* (556433; BD Biosciences); Histone H3 (4499; Cell Signalling); Tom20 (sc-17764; Santa Cruz); Tom22 (sc-58308; Santa Cruz); Tim50 (22229-1-AP; Proteintech); Tim23 (611222; BD Biosciences); Tim44 (138-59-1-AP; Proteintech); Tim29 (HPA041858; Sigma); Mic60 (sc-390707; Santa Cruz); SDHA (ab14715; Abcam); VDAC1 (sc-390996; Santa Cruz); LAMP1 (H4A3-C; DSHB); PDI (ADI-SPA-891-D; Sapphire Bioscience); Pex14 (10594-1-AP; Proteintech); and OPA1 (612606; BD Biosciences). Antibodies against Bak, NDUFAF2, NDUFA9, Mfn2, and Sam50 were generously provided by Prof. Mike Ryan (Monash University).

## Results

### Purification of Mitochondria From *C. burnetii*-Infected Cells

To identify effector proteins targeted to the mitochondria during *C. burnetii* infection, an unbiased, unlabeled, proteomic approach was employed. An experimental pipeline to obtain pure mitochondria from *C. burnetii*-infected cells for downstream proteomic analysis was required. The purity of this sample was vital to aid in the identification of high-confidence mitochondrial-associated effector proteins, as contamination with *C. burnetii* would result in the misidentification of translocated proteins. Additionally, contamination of mitochondria with other host cell organelles could decrease the mitochondrial specificity of the identified effector proteins. A standard mitochondrial isolation, using differential centrifugation, yields “crude” mitochondria with a low degree of purity. We therefore opted for a more stringent approach based on published methods (45, 60, 61, 62, 63). As these published methods had not been performed in the context of bacterial infection, we asked if one, or a combination, of these approaches could be applied to purify mitochondria from *C. burnetii*-infected cells.

**Fig. 1:**
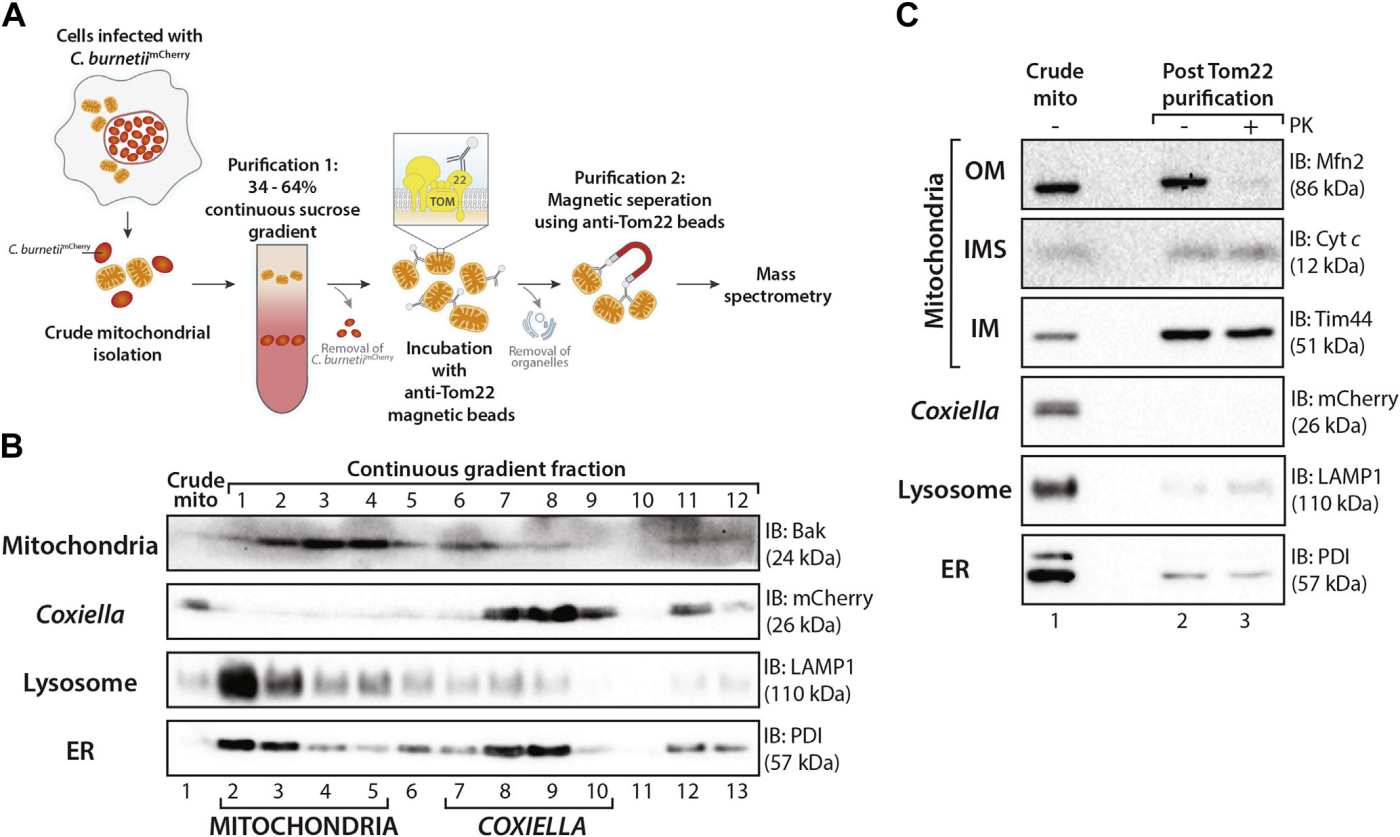
**Mitochondrial purification from *C. burnetii*-infected cells.***A*, schematic representation of mitochondrial purification method from *C. burnetii*-infected cells. *B*, continuous sucrose gradient centrifugation of mitochondria isolated from *C. burnetii*^mCherry^-infected HeLa cells. A crude mitochondrial sample was taken pre-centrifugation (crude mito). Fractions were collected following ultracentrifugation and analyzed by SDS-PAGE and immunoblotting with the indicated antibodies. Fractions containing the majority of mitochondrial or *C. burnetii* protein are marked with “mitochondria” or “*Coxiella*”, respectively. *C*, mitochondria were isolated from THP-1 cells infected for 3 days with *C. burnetii*^mCherry^ and purified by continuous sucrose gradient centrifugation and anti-Tom22 separation. Samples were collected after crude mitochondrial isolation (Crude mito, lane 1) and after purification (Post Tom22 purification, lanes 2 and 3). Purified mitochondria were incubated with or without Proteinase K (PK; 50 μg/ml) and analyzed by SDS-PAGE and immunoblotting using the indicated antibodies as the listed organelle markers. Cyt *c*, cytochrome *c*; IMM, inner mitochondrial membrane; IMS, intermembrane space; ER, endoplasmic reticulum; OMM, outer mitochondrial membrane; THP-1, human monocyte–derived macrophage cell line.

Mitochondria were isolated by differential centrifugation from HeLa cells persistently infected with *C. burnetii* constitutively expressing mCherry (*C. burnetii*^mCherry^), which functioned as a downstream marker for bacterial detection. The crude mitochondrial sample was overlaid on a 34 to 64% (w/v) continuous sucrose gradient and separated by ultracentrifugation (Fig. 1*A*). Mitochondria previously isolated by this method were recovered at a sucrose density of ∼40% (w/v) (45). Fractions were collected, and proteins were separated by SDS-PAGE to analyze organelle and bacterial content (Fig. 1*B*). Mitochondria and *C. burnetii* separated into distinct fractions of the continuous gradient (Fig. 1*B*, “mitochondria” lanes 3–6 and “*Coxiella*” lanes 8–10); however, fractions in which mitochondrial protein was recovered contained other organelles, demonstrated by markers for the lysosomes and the endoplasmic reticulum (ER) (Fig. 1*B*, “ER” and “Lysosomes”). Thus, separation of crude mitochondria on a sucrose gradient was effective at separating *C. burnetii* and mitochondria; however, further purification was required to remove host cell contamination. To do this, we employed magnetically conjugated monoclonal antibodies directed against human Tom22 (hTom22), a receptor protein of the translocase of the outer mitochondrial membrane (TOM) complex (63), to separate mitochondria from remaining cellular organelles.

Once a purification pipeline was established in HeLa cells, we moved to a system that more closely resembled the host cell environment during infection and shifted to using differentiated human monocyte–derived macrophage cell line (THP-1) cells for all further experiments. *C. burnetii* infects and replicates within both phagocytic and nonphagocytic cells without any gross changes to mitochondrial morphology (supplemental Fig. S1). THP-1 cells were infected with *C. burnetii* for 3 days, and mitochondria were isolated by differential centrifugation, followed by purification on a continuous sucrose gradient and then magnetic Tom22 affinity purification. Mitochondria subjected to this purification (“post Tom22 purification”) had an enrichment of mitochondrial proteins and a decrease in abundance of *C. burnetii* as indicated by lack of mCherry signal in purified sample (Fig. 1*C* “*Coxiella*”) and less lysosome and ER contamination (Fig. 1*C* “lysosomes” and “ER”). Addition of external protease, Proteinase K (PK), to assess the integrity of the mitochondrial outer membrane resulted in loss of detectable signal of the outer membrane protein Mfn2, whereas both cytochrome c (intermembrane space [IMS] localized) and Tim44 (inner membrane localized) were protected, indicating organelle integrity was maintained (Fig. 1*C*, lane 3).

### Proteomic Analysis of Purified Mitochondria From Infected Cells Reveals Depletion of *C. burnetii* Proteins and Association of *C. burnetii* Proteins With Mitochondria

**Fig. 2:**
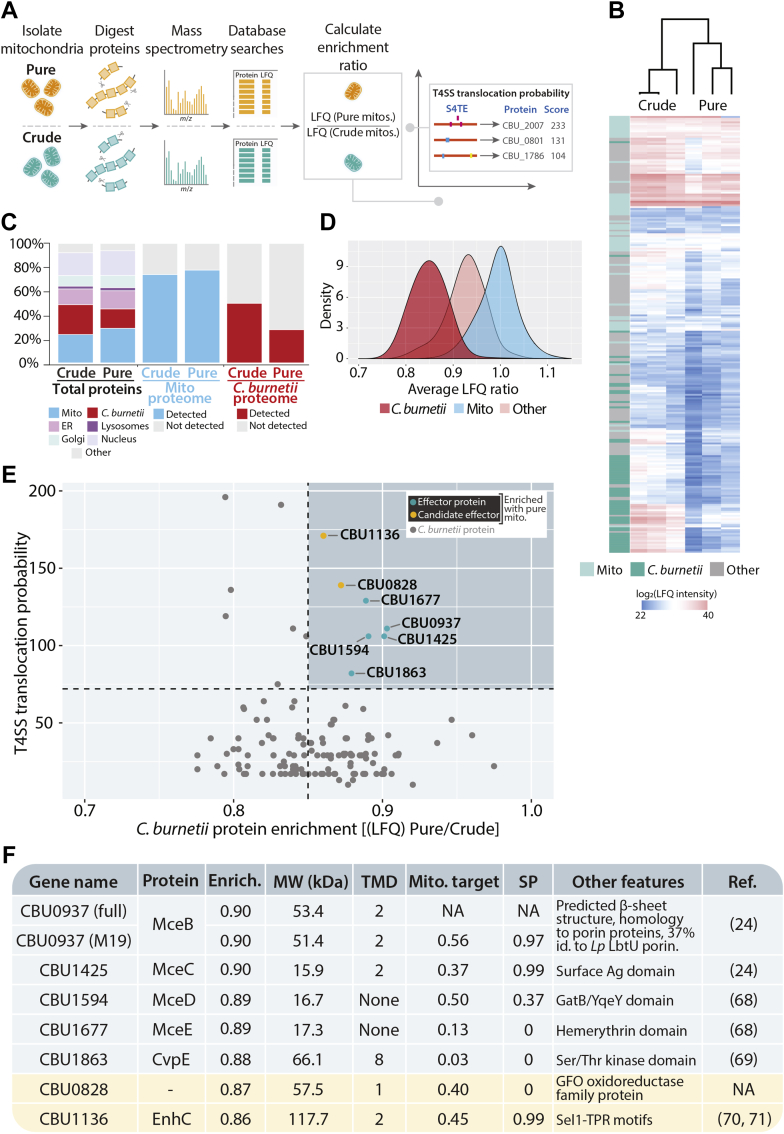
**Proteomic analysis of mitochondria isolated from *C. burnetii*-infected THP-1 cells.***A*, overview of proteomic analysis pipeline. “Crude” and “Pure” mitochondrial samples were analyzed by label-free quantitative (LFQ) mass spectrometry. LFQ values of individual proteins were compared to calculate the enrichment ratio. The enrichment ratio was then compared with the probability that a *C. burnetii* protein had of being an effector (T4SS score; https://sate.cirad.fr). *B*, heatmap and cluster analysis of “Crude” and “Pure” proteomics samples. Mitochondrial proteins are labeled in *light teal*, *C. burnetii* proteins are labeled in *green*. n = 3 biological replicates per “crude” and “pure” fraction. Heatmap key denotes LFQ value (range 20–40). *C*, analysis of proteome coverage. Left columns: Total proteins identified across three biological replicates were classed as *C. burnetii* (*red*; *C. burnetii* proteome), mitochondrial (*blue*; MitoCarta 2.0 annotations), or endoplasmic reticulum (ER; *pink*), Golgi apparatus (Golgi; *turquoise*), or lysosomal (Lysosomes; *purple*) according to Gene Ontology Cellular compartment annotation. Remaining proteins outside these categories were labeled “other” (*gray*). Each number was compared to the total number of proteins identified in both the crude and pure mitochondrial preparations. Middle columns: Total mitochondrial proteins detected in crude and pure preparations were compared with the MitoCarta 2.0 mitochondrial proteome and coverage depicted. Right columns: Total *C. burnetii* proteins detected in crude and pure fractions were compared with the annotated *C. burnetii* proteome. *D*, density plot depicting the distribution of LFQ enrichment ratio (calculated as in [*A*]). Enrichment ratio (LFQ in Pure/LFQ in Crude) of mitochondrial proteins (*blue*) and *C. burnetii* proteins (*red*) shown. Remaining proteins represented in *pink*. *E*, scatter plot of *C. burnetii* protein enrichment factor (horizontal axis: LFQ [Pure/Crude]) compared with the probability of translocation by the T4SS (vertical axis). Dotted lines represent cut-offs: T4SS score ≥72; enrichment ratio >0.85. 0.85 was selected as a cut-off due to it being the mean of the distribution curve from *Coxiella* proteins, after which the amount of *C. burnetii* proteins with a high enrichment ratio began to decline. Proteins with a high enrichment ratio and high probability of translocation shown in top right (*blue quadrant*). Proteins known to be translocated into the host cell are shown in *green* (“effector protein”), proteins currently unannotated as effector proteins shown in yellow (“candidate effector”). All other *C. burnetii* proteins depicted in *gray*. *F*, bioinformatic analysis of enriched *C. burnetii* proteins identified in top right quadrant of (*A*). “Enrich.”, enrichment ratio; Evidence/ref., evidence/reference of T4SS-translocation or previously published studies regarding the protein; Mito. Target, mitochondrial targeting signal prediction (determined using Mitoprot); MW (kDa), molecular weight (kilodaltons); other features, additional features/homology to other proteins (determined using HHpred); SP, signal peptide prediction; THP-1, human monocyte–derived macrophage cell line; TMD, transmembrane domain; T4SS, type IV secretion system.

Mitochondria were isolated from *C. burnetii*-infected differentiated THP-1 cells at 3 days post-infection and purified as described in Figure 1. Equal amounts of sample were reserved at both the initial isolation and following the purification, designated “crude” and “pure” respectively, and analyzed by label-free quantitative mass spectrometry (Fig. 2*A*). Human and *C. burnetii* proteome coverage was assessed, and proteins retained in the final dataset were identified in at least two of the three replicates analyzed, the combined results of which identified >3000 proteins (supplemental Table S1). Distinct proteome profiles were evident on comparing crude and pure replicates (Fig. 2*B*). Encouragingly, proteins identified as bacterial showed a reduction in abundance in the pure mitochondrial sample compared with the crude (Fig. 2*B*, *C. burnetii* proteins highlighted in *dark green*). Mitochondrial proteins were defined based on the human mitochondrial proteome database, MitoCarta (Fig. 2*B*, supplemental Table S1, “Mitochondrial”) (55, 56). Quantitative comparison of the label-free quantitation (LFQ) value of individual proteins within the crude and pure samples revealed the extent of the *C. burnetii* proteome coverage decreased ∼40% following purification confirming this method as valid in minimizing *C. burnetii* present in the final mitochondrial sample (Fig. 2*C*). Mitochondrial proteome coverage was moderately increased between the crude and pure samples (857 proteins listed in the human MitoCarta 2.0 mitochondrial database detected in crude, 900 detected in pure), indicating the purification technique was not detrimental to the organelle integrity (Fig. 2*C*) (55). Gene ontology analysis for cellular compartments revealed proteins classed as neither mitochondrial nor bacterial were identified in both the crude and pure samples. These proteins were annotated as residing in the ER, lysosomes, or Golgi apparatus (Fig. 2*C*). It is plausible that some of these proteins are from compartments in close association or contact with the mitochondrion such as the mitochondrial-associated membrane. Importantly, many non-mitochondrial proteins were depleted in the pure sample compared with the crude, thus confirming the method as a robust approach to purify mitochondria from *C. burnetii*-infected cells.

To determine the extent of protein enrichment as a result of purification, the LFQ values of host cell and bacterial proteins in the pure mitochondrial sample were compared to the crude, for each biological replicate (Fig. 2*A*, supplemental Table S1: “LFQ pure/LFQ crude”). This ratio was then averaged across biological replicates to yield a final “enrichment ratio” (Fig. 2*A*, supplemental Table S1: “LFQ ratio Average”). This revealed higher LFQ ratios among mitochondrial proteins and therefore greater enrichment of mitochondrial proteins compared with *C. burnetii* proteins within the pure dataset (Fig. 2*D*, compare mitochondria [blue] to *C. burnetii* [red]). These analyses indicate that the dual purification method enriches for mitochondrial proteins while decreasing the abundance of *C. burnetii* proteins within the pure sample.

Despite the rigorous method of purification and the reduction in co-purification of *C. burnetii* with mitochondria, bacterial proteins were still detected within the pure mitochondrial fraction (Fig. 2*C*), showcasing both the sensitivity and challenges of mass spectrometry approaches. To distinguish between contaminating *C. burnetii* proteins and effector proteins, we used the S4TE 2.0 Type 4 effector protein predictive tool (https://sate.cirad.fr) to generate a probability score of a *C. burnetii* protein being an effector protein (57). Analysis of the *C. burnetii* genome by the S4TE program generated a list of 1818 proteins with an assigned score of translocation probability between 0 and 233 with a proposed effector score threshold >72 (supplemental Table S2) (57). On comparison to the established cohort of 150 *C. burnetii* effector proteins, the S4TE output scores identified the majority of effector proteins although open reading frames unannotated in the National Centre for Biotechnology gene, nucleotide, or protein database received either no score or a score below 72. The score for these proteins was manually annotated to 75 to account for this discrepancy (supplemental Table S2). To identify *C. burnetii* effector proteins associated with the mitochondria during infection, the enrichment factor was compared with the S4TE output (termed “T4SS probability”) (Fig. 2*A*, supplemental Table S2). To ensure a high confidence list of proteins was produced, strict cut-offs for both parameters were applied: *C. burnetii* proteins were considered with an enrichment factor greater than 0.85, as this was above the mean of the distribution of *C. burnetii* proteins enrichment ratio (Fig. 2*D*) and T4SS probability score greater than 72 to ensure a high-certainty of effector protein prediction. A cluster of seven bacterial proteins were enriched alongside isolated mitochondria and had a high probability of being translocated by the T4SS (Fig. 2*E*, top right quadrant). MceA (CBU0077), a known effector protein targeted to mitochondria (28), was identified in crude and purified mitochondria (supplemental Table S1), but a low number of peptide sequences associated with the protein in the purified samples did not culminate in valid LFQ values resulting in the proteins removal from the final dataset (supplemental Table S1). Nevertheless, identification of unique peptides from MceA in purified mitochondria confirms this technique as a valid approach to identify candidate mitochondrial-targeted effector proteins. The seven identified *C. burnetii* proteins exhibited a range of predicted biochemical properties, with diversity in size, predicted transmembrane domains (TMDs), and the presence of signal peptide or putative mitochondrial targeting signals (Fig. 2*F*). In addition to five effector proteins known to be translocated into the host cell (24, 64, 65) (Fig. 2*E*, proteins shown in green), our proteomics data revealed an additional two proteins that may represent candidate effector proteins (Fig. 2*E*, proteins shown in yellow). CBU1136 encodes multiple Sel-1-type TPR motifs and annotated as enhanced entry protein C due to homology to the *L. pneumophila* protein of the same name (66). Although, CBU1136 and CBU0828 represent exciting candidates for future research, we focused on the established effector proteins: CBU0937, CBU1425, CBU1594, and CBU1677, as CBU1863 has been previously characterized elsewhere (65).

### Confirmation of the Subcellular Localization of Mitochondrial-Associated Effector Proteins

**Fig. 3:**
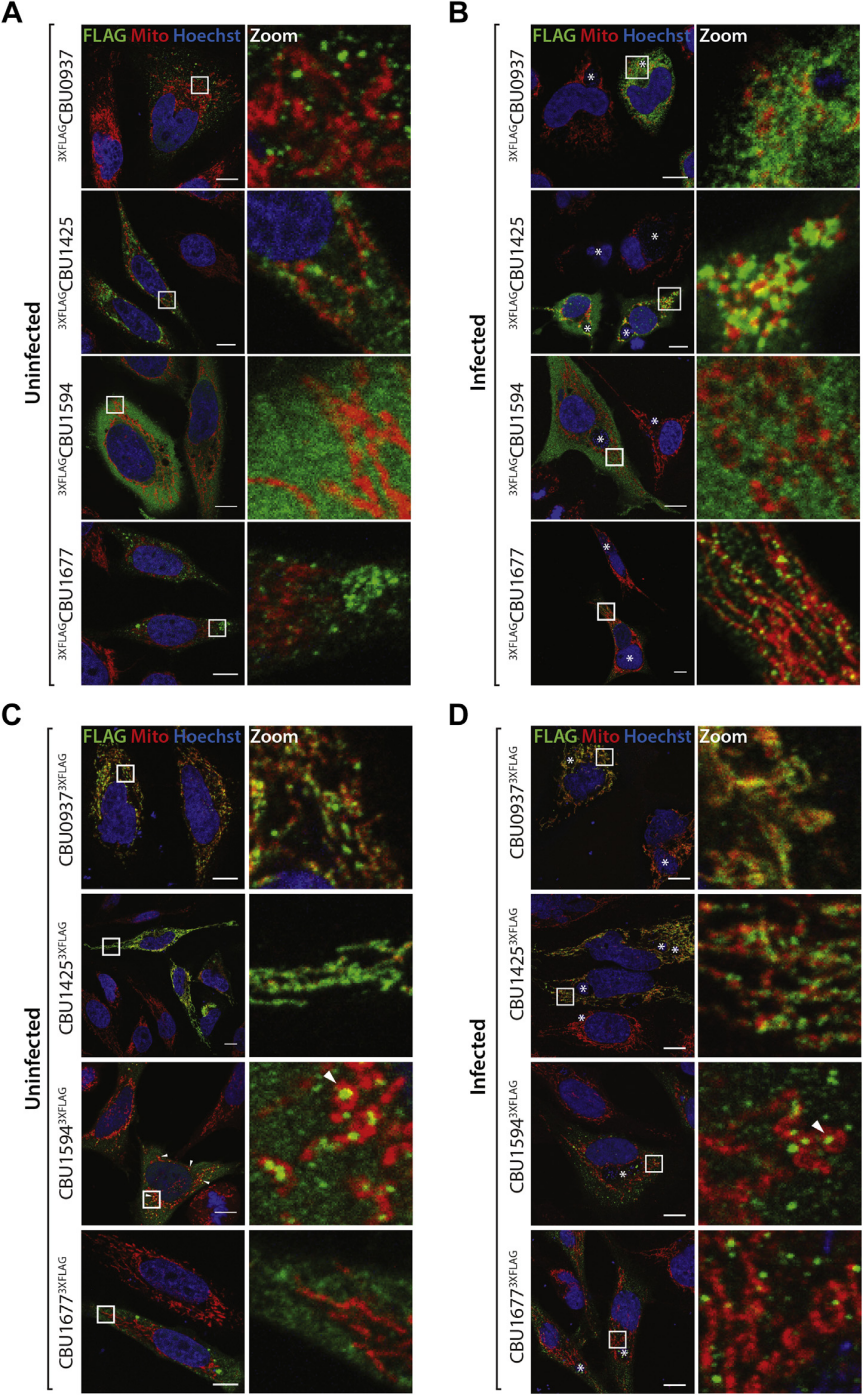
**Localization of N-terminally and C-terminally tagged, novel mitochondrial-targeted *C. burnetii* effector proteins.***A*, uninfected HeLa cells were transfected for 18 h with N-terminally 3XFLAG-tagged effector protein. *B*, *C. burnetii*-infected HeLa cells were transfected for 18 h with N-terminally 3XFLAG-tagged effector protein. *C*, uninfected HeLa cells were transfected for 18 h with C-terminally 3XFLAG-tagged effector protein. *D*, *C. burnetii*-infected HeLa cells were transfected for 18 h with C-terminally 3XFLAG-tagged effector protein. All cells were fixed and immunostained with antibodies against FLAG (*green*) and NDUFAF2 (Mito; *red*). Nucleus was stained with Hoechst 33,258 (*blue*). Cells were imaged by confocal microscopy. Scale bar represents 10 μm. Right panels show a magnified view of the boxed region (“zoom”). *Asterisks* denote position of CCV. *Arrowheads* denote regions of interest. CCV, *Coxiella*-containing vacuole.

We complemented the proteomic data with the ectopic expression of the effector proteins and fluorescence microscopy to provide further evidence of their subcellular localization. Ectopic expression of effector proteins also supports downstream biochemical analysis, as native effector proteins are often expressed at very low levels in host cells. The selected effector proteins (CBU0937, CBU1425, CBU1594, and CBU1677) were epitope tagged (3XFLAG) at N or C termini and expressed in HeLa cells, since the monocyte-derived THP-1 cells exhibit an immune response to the introduction of plasmid DNA via transfection. We took into consideration that addition of the peptide tag may occlude targeting signals within the effector protein as well as the chance that additional effector proteins that are present during infection may act as chaperones to aid in effector targeting (30, 67). Therefore, the N- and C-terminally tagged effectors were expressed in both uninfected and *C. burnetii*-infected HeLa cells (persistent infection). Transfections were performed for 18 h to capture protein localization before gross overexpression. The host cell mitochondrial network was identified by staining for the matrix localized protein NDUFAF2, a Complex I assembly factor. Expression of 3XFLAG fusion proteins in HeLa cells revealed a diversity in localization patterns. The position of the 3XFLAG-tag influenced the localization of the expressed proteins. Effector proteins with a 3XFLAG-tag located at the N-terminus of the protein, with the exception of CBU1425, did not demonstrate any clear co-localization with the mitochondrial network (Fig. 3*A*). A partial association with mitochondrial tubules was observed for ^3XFLAG^CBU1425 (Fig. 3*A*). The targeting of N-terminally tagged effector proteins was then assessed in the context of *C. burnetii* infection (Fig. 3*B*, *Coxiella*-containing vacuole marked by ∗). Consistently, ^3XFLAG^CBU1425 localized in the vicinity of mitochondria (Fig. 3*B*), while localization differences were observed for ^3XFLAG^CBU1677 (Fig. 3*B*). ^3XFLAG^CBU1677 expressed in uninfected cells was identified in vesicle-like structures located around the nucleus and condensed at opposing poles of the cell (Fig. 3*A*). However, during *C. burnetii*-infection, transfected ^3XFLAG^CBU1677 appeared to align with mitochondrial tubules in semi-regularly spaced foci (Fig. 3*B*).

Next, the localization of effector proteins tagged at the C-terminus was established. The altered position of the 3XFLAG tag had a profound effect on the observed localization of CBU0937, CBU1425, and CBU1594 (Fig. 3*C*). In contrast to the absence of any distinct localization of ^3XFLAG^CBU0937, CBU0937^3XFLAG^ co-localized with the host cell mitochondrial network, distributed along mitochondrial tubules (Fig. 3*C*). Similarly, CBU1425^3XFLAG^ was also found to co-localize with the signal from the mitochondrial marker NDUFAF2 (Fig. 3*C*). CBU1594^3XFLAG^ localized to distinct, punctate structures that were in the vicinity of mitochondria (Fig. 3*C*). Intriguingly, these CBU1594^3XFLAG^ puncta were often found in the middle of mitochondrial tubules that had curved into donut-shaped structures (Fig. 3*C*, white arrowheads). CBU1677^3XFLAG^ demonstrated a similar localization pattern as the N-terminally tagged equivalent (^3XFLAG^CBU1677) in uninfected cells, with concentrations of protein near to the nucleus (Fig. 3*C*).

Localization of C-terminally tagged effector proteins in *C. burnetii*-infected cells revealed, as for uninfected cells, CBU0937^3XFLAG^ and CBU1425^3XFLAG^ co-localized with the mitochondrial network (Fig. 3*D*, *Coxiella*-containing vacuole marked by ∗). CBU1594^3XFLAG^ was again located in punctate structures close to mitochondria, occasionally encircled within mitochondrial tubules (Fig. 3*D*, white arrowheads). Similar to the N-terminally tagged protein expressed in infected cells, CBU1677^3XFLAG^ localized to vesicular structures in close association with mitochondria, indicating that the targeting information for this protein possibly requires additional factors (presumably other effector proteins) only present during infection (Fig. 3*D*).

Taken together, the findings of our proteomic screen identified seven novel proteins associated with the mitochondrion during infection, and microscopic analysis of four of these proteins supports their mitochondrial localization: CBU0937, CBU1425, CBU1594, and CBU1677. We therefore propose to name these proteins mitochondrial *Coxiella* effector proteins B (CBU0937), C (CBU1425), D (CBU1594), and E (CBU1677). These proteins display some variation in localization at the mitochondria, from a more uniform distribution along the mitochondrial tubules as for MceB and MceC to more punctate structures such as MceD and MceE (Fig. 3). Variation in targeting of these proteins was observed dependent on the location of the tag and the context of infection, providing valuable insight into the targeting information required for protein localization.

### Characterizing the Role of MceC—A *C. burnetii* Effector Protein Targeted to the Mitochondria During Infection

We decided to investigate MceC further as this protein displayed a striking localization with mitochondria. MceC is a 15.9 kDa protein, predicted to contain two TMDs toward the N-terminus (supplemental Fig. S2*A*). Sequence analysis of MceC revealed the protein contains multiple glycine zipper (GXXXG or GGXXG) motifs spanning residues 27 to 69 and encompassing the two TMDs (supplemental Fig. S2*A*). Glycine-zipper motifs are often found in TMDs and typically assist in mediating protein–protein interactions such as homo-oligomerization and helix packing within a membrane (68). The C-terminal part of the protein contains a region with 42% homology to a “17 kDa outer membrane surface antigen” domain present in some Proteobacterial species. BLAST searches do not reveal significant homology to any mammalian proteins, which is common for *C. burnetii* effector proteins. Earlier studies focused on uncovering *C. burnetii* T4SS substrates demonstrated MceC was delivered into the host cell by the T4SS using a BlaM reporter assay (24). The subcellular localization of the protein has been previously suggested to be mitochondrial; however, no experimental evidence for this phenotype was provided (64).

**Fig. 4:**
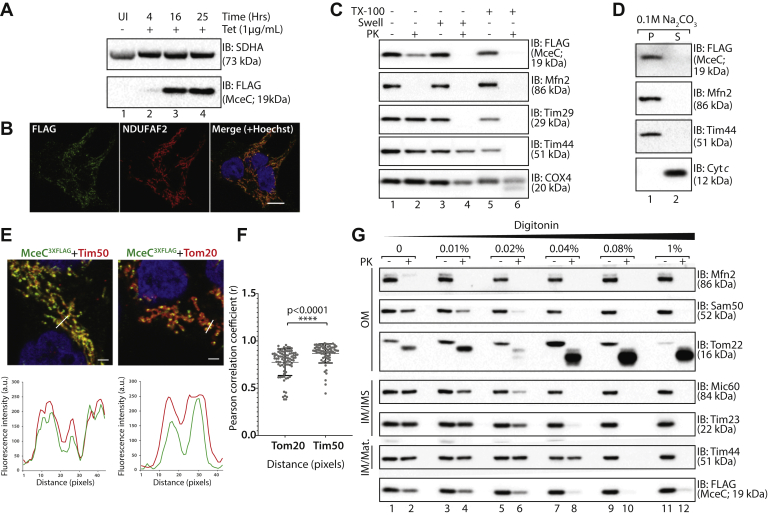
**Characterizing the submitochondrial localization of MceC.***A*, protein expression in tetracycline inducible HEK293^MceC-3XFLAG^ cells. Cells were left untreated or treated with tetracycline (1 μg/ml) for 4, 16, or 25 h to induce expression of MceC^3XFLAG^. Cells were collected and analyzed by SDS-PAGE and immunoblotting with the indicated antibodies. *B*, HEK293^MceC-3XFLAG^ cells were treated for 16 h with tetracycline (1 μg/ml) before being fixed and stained with antibodies against FLAG (*green*) and NDUFAF2 (Mito; *red*). Nucleus was stained with Hoechst 33258 (*blue*). Scale bar represents 10 μm. *C*, mitochondria were isolated from HEK293 cells expressing MceC^3XFLAG^ and either left intact, (lanes 1 and 2), subjected to hypotonic swelling (“Swell”) of the outer mitochondrial membrane (mitoplasts; lanes 3 and 4) or solubilized mitochondria (TX-100; lanes 5 and 6). Samples were left untreated or incubated with Proteinase K (PK; 50 μg/ml, lanes 2, 4, and 6) before analysis by SDS-PAGE and immunoblotting with the indicated antibodies. *D*, mitochondria isolated from HEK293 cells expressing MceC^3XFLAG^ were subjected to alkaline extraction in sodium carbonate (Na_2_CO_3_; 0.1 M, pH 11). Following incubation on ice, membrane (P) and soluble (S) fractions were separated by ultracentrifugation prior to SDS-PAGE and immunoblotting with the indicated antibodies. *E*, HEK293^MceC-3XFLAG^ cells were treated for 16 h with tetracycline (1 μg/ml) before being fixed and immunodecorated with antibodies against FLAG (*green*) and mitochondrial outer membrane protein Tom20 or mitochondrial inner membrane protein Tim50 (*red*). The nucleus was stained with Hoechst (*blue*). Scale bar represents 10 μm. Representative line scan location demonstrated below each fluorescence profile, scale bar represents 2 μm. Relative fluorescence intensities were measured. *F*, Pearson's coefficient of correlation between MceC^3XFLAG^ and Tom20 or Tim50 fluorescence, ∗∗∗∗ *p*-value < 0.0001. *G*, mitochondria isolated from cells expressing MceC^3XFLAG^ were solubilized in the indicated concentrations of digitonin and treated with Proteinase K. Samples were processed for SDS-PAGE and immunoblotting with the indicated antibodies. IM, inner membrane; IMS, intermembrane space; Mat., matrix; OM, outer membrane.

A stable tetracycline-inducible HEK293T cell line expressing MceC^3XFLAG^ was created, protein expression induced for 4, 16, and 25 h, and expression was analyzed by immunoblotting (Fig. 4*A*). As early as 4 h, MceC^3XFLAG^ could be detected, and a steady increase in expression was evident, while little to no change was apparent in the levels of the loading control, the Complex II subunit SDHA (Fig. 4*A*). At 16 h induction, MceC^3XFLAG^ was observed to co-localize with the mitochondrial marker NDUFAF2, confirming the expression of the protein detected by immunoblotting and correct protein targeting (Fig. 4*B*). As the levels of bacterial effector proteins are typically very low within cells, it was important to determine that the level of MceC expression was not causing protein misfolding and/or aggregation. Solubility trials using different detergents (TX-100, DDM, or digitonin) were performed on mitochondria isolated at 4 and 8 h post-tetracycline induction. Total, pellet, and supernatant fractions were analyzed by SDS-PAGE and immunoblotting and showed MceC^3XFLAG^ was recovered in the supernatant fraction following 4 h of induction (supplemental Fig. S2*B*, lanes 3, 6, 9, and 12). Samples induced for 8 h displayed the protein in the pellet during the digitonin condition, indicating the protein was insoluble (supplemental Fig. S2*C*); therefore, protein expression for 4 h was selected for further experiments.

We assessed which mitochondrial subcompartment MceC^3XFLAG^ was localized within by performing a mitochondrial subfractionation assay. Mitochondria isolated from HEK293 expressing MceC^3XFLAG^ cells were left untreated, subjected to swelling in hypo-osmotic buffer or completely solubilized in TX-100 (Fig. 4*C*). The accessibility of proteins in each of these conditions to PK was then assessed before analysis by SDS-PAGE and immunodecoration for proteins of distinct mitochondrial subcompartments. The FLAG signal, corresponding to MceC^3XFLAG^, was inaccessible to PK in intact mitochondria; however, protection was lost following the rupture of the outer membrane during swelling (Fig. 4*C*, compare lanes 2 and 4). This pattern of degradation is similar to the inner membrane protein Tim29, a component of the TIM22 complex, but distinct from the inner membrane/matrix Complex IV subunit, COX4 (Fig. 4*C*). These results indicate that the C-terminus (location of 3XFLAG-tag) of MceC^3XFLAG^ is located within the IMS of mitochondria. We noted a drop in the intensity of FLAG signal upon treatment of intact mitochondria with PK (Fig. 4*C*, compare lanes 1 and 2) but attributed this to the accumulation of pre-imported MceC^3XFLAG^ at the outer membrane, as the expression level in the inducible cell system, while regulated, is still higher than the endogenous levels of protein during infection. As MceC is predicted to contain two TMDs, the integration of the protein into a mitochondrial membrane was assessed using carbonate extraction. Mitochondria isolated from HEK293 cells expressing MceC^3XFLAG^ were resuspended in sodium carbonate for separation of membrane proteins and peripheral membrane proteins by high-speed centrifugation (Fig. 4*D*). MceC^3XFLAG^ was localized to the pellet fraction, alongside Mfn2 and Tim44, indicating that the protein is indeed integrated into a mitochondrial membrane with the C-terminus in the IMS.

The data indicated MceC could be located to either the outer or inner membrane while still maintaining the C-terminus in the IMS and thus the 3XFLAG-tag accessible for degradation. To examine this further, we used both fluorescence microscopy and further biochemical characterization of isolated mitochondria. Immunofluorescence microscopy was performed on cells expressing MceC^3XFLAG^ and colocalization with the outer membrane receptor Tom20 or the inner membrane localized Tim50 assessed (supplemental Fig. S2*D*). MceC^3XFLAG^ and Tim50 had a greater overlap of fluorescence signal and this was verified using Pearson's correlation analysis that showed a significantly higher coefficient of correlation with Tim50 than Tom20 (Fig. 4, *E*–*F*). These data suggested that MceC is localized to the inner membrane and this was further verified biochemically by subjecting mitochondria isolated from HEK293 cells expressing MceC^3XFLAG^ to digitonin solubilization and protease protection analysis (Fig. 4*G*). Consistent with our mitochondrial subfractionation analysis, an amount of MceC was accessible to degradation by PK on treatment of intact mitochondria (Fig. 4*G*, lane 2). However, the levels of MceC protected from protease degradation remained consistent despite solubilization of the outer mitochondrial membrane with low concentrations of digitonin (Fig. 4*G*, compare lanes 2 and 4). This was in contrast to outer mitochondrial membrane proteins Mfn2, Tom22, and Sam50, all of which demonstrated protease susceptibility in intact mitochondria and on the addition of 0.01% (w/v) digitonin (Fig. 4*G*, lanes 1–4). MceC displayed a pattern of protease protection more closely resembling that of the inner membrane localized, Tim23 and Mic60 (Fig. 4*G*, lanes 3 and 4). Taken together, these data confirm that MceC is imported into the mitochondria and localizes to the inner mitochondrial membrane (IMM) (supplemental Fig. S2*E*).

### MceC Interacts With Components of the Mitochondrial Quality Control Machinery

**Fig. 5:**
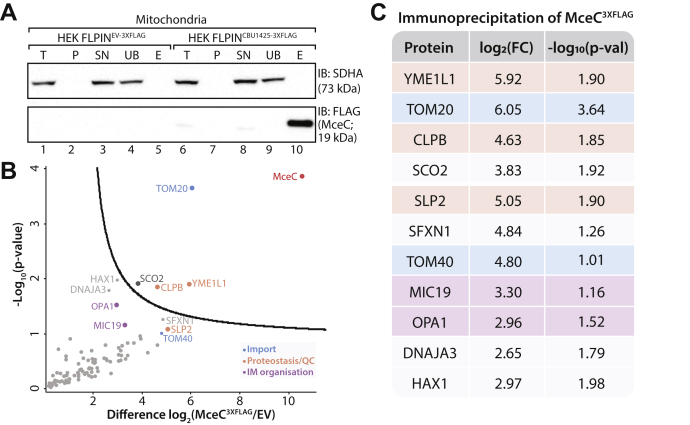
**MceC immunoprecipitates resident mitochondrial proteins.***A*, mitochondria isolated from control and HEK293 cells expressing MceC^3XFLAG^ for 4 h were solubilized in digitonin (1% [w/v])-containing buffer and lysates subjected to anti-FLAG immunoprecipitation. Collected fractions were analyzed by SDS-PAGE and immunoblotting with the indicated antibodies. 10% of the T, P, SN, and UB fractions and 100% of the elution (E) fraction was loaded for analysis. *B*, volcano plot showing proteins enriched following MceC immunoprecipitation as outlined in (*A*) compared with control “empty vector” (EV) cells. Mitochondrial proteins plotted and each circle represents one protein. Gene names of selected proteins are used for labels. Horizontal axis shows the Log_2_(fold change) of MceC interacting partners and vertical axis shows -Log_10_(p-value) of two-sample Student's *t*-test (FDR: 0.05, s0: 1). n = 3 biological replicates. Colors correspond to key. *C*, proteins identified by quantitative mass spectrometry following immunoprecipitation of MceC in (*B*). Gene names of selected proteins used as protein label. Corresponding Log_2_(fold change) and Log_10_(p-value) of each protein listed. Colors as per key in (*B*). E, elution; IM, inner membrane; P, pellet; QC, quality control; SN, supernatant; UB, unbound; T, total.

Effector proteins typically act to modulate a specific host process. This often involves a direct interaction with host cell proteins. We sought to identify any mitochondrial interacting partners of MceC by immunoprecipitation of MceC^3XFLAG^. MceC^3XFLAG^ and associated proteins were purified by FLAG antibodies and eluates separated on SDS-PAGE to confirm efficiency of pull-down (Fig. 5*A*). A signal corresponding to MceC^3XFLAG^ was detected using FLAG antibodies in the elution fraction of HEK293^MceC-3XFLAG^ cells but not control cells (Fig. 5*A*, lanes 5 and 10). Accordingly, the mitochondrial protein SDHA was not detected in the elution (Fig. 5*A*, lane 10). When eluates from three independent, biological replicates were processed for label-free quantitative mass spectrometry, we observed a successful enrichment of MceC^3XFLAG^ in addition to several other mitochondrial proteins (Fig. 5, *B–C* and supplemental Table S3). Intriguingly, these proteins included important players in the mitochondrial proteostasis and quality control pathways YME1L, SCO2, caseinolytic peptidase B (CLPB) and SLP2 as well as components of the TOM complex: TOM20 and TOM40 (Fig. 5, *B–C* and supplemental Table S3). Additionally, key proteins involved in inner membrane and cristae morphology, OPA1, and the MICOS subunit MIC19 were also enriched although not above the significance threshold (Fig. 5, *B*–*C* and supplemental Table S3).

To assess if any of these interactions were conserved during infection, HEK293^MceC-3XFLAG^ and control cells were persistently infected with *C. burnetii* before protein induction. Mitochondria were isolated, solubilized in digitonin, and immunoprecipitation with FLAG antibodies performed before analysis by mass spectrometry. Consistent with the interactions observed in uninfected cells, SLP2 was enriched alongside MceC^3XFLAG^ in mitochondria isolated from infected cells (supplemental Fig. S3 and supplemental Table S4). Interaction with YME1L was not observed in infected cells and may indicate CBU1425 interaction with the protease is less stable during infection.

### Impact of MceC Expression on Mitochondrial Function

**Fig. 6:**
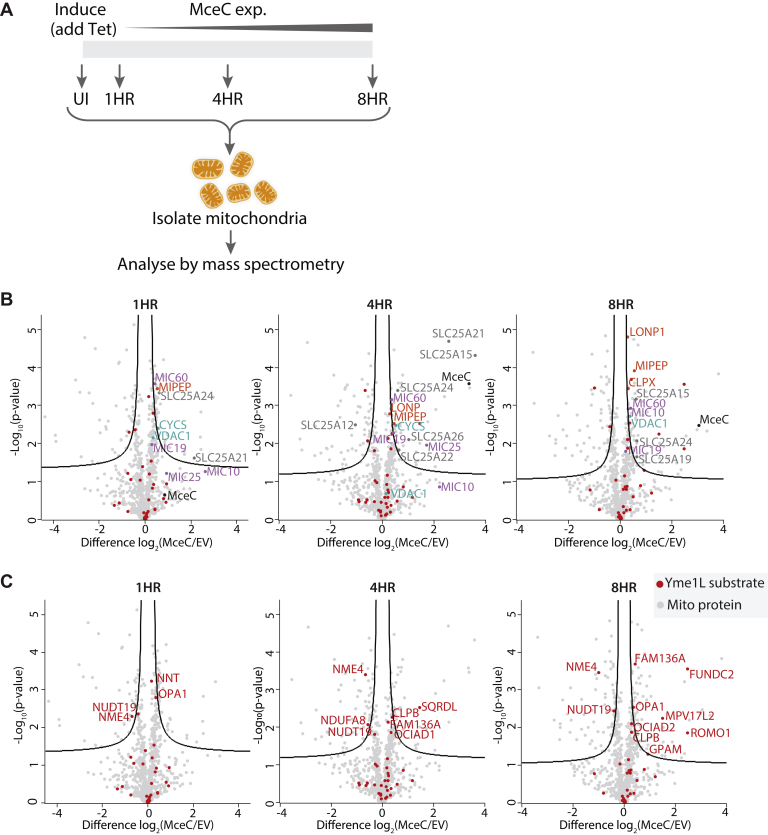
**Expression of MceC affects mitochondrial function.***A*, experimental pipeline for label-free quantitative proteomics analysis of mitochondria isolated from control (EV) or MceC-expressing cells following 1, 4, or 8 h tetracycline-induction. n = 3 biological replicates per timepoint. *B*, volcano plots showing relative abundance of proteins in mitochondria isolated from MceC-expressing cells compared with control (EV) cells. Significantly altered proteins were identified using a Student's *t*-test with an FDR of 0.05 and S0 of 0.1. Proteins of interest are indicated. Gene names of selected proteins used as labels. *C* volcano plots showing relative abundance of proteins in mitochondria isolated from MceC-expressing cells compared with control (EV) cells. Significantly altered proteins were identified using a Student's *t*-test with an FDR of 0.05 and S0 of 0.1. Known YME1L substrate proteins are labeled in *red*. Gene names of selected proteins of interest used as labels. EV, empty vector; FDR, false discovery rate.

To gain perspective on the wider implications of MceC localization to the mitochondria, we looked more broadly at changes to the mitochondrial proteome over an expression time-course of the effector protein. Label-free quantitative mass spectrometry was performed on mitochondria isolated following 1, 4, and 8 h MceC^3XFLAG^ expression (Fig. 6*A*). Triplicate samples were compared using an ANOVA test to determine significantly altered protein abundance at each time point. This revealed 189 proteins with a false discovery rate (FDR) of 5% and S0 of 0 (supplemental Table S5). These 189 proteins were involved in a range of mitochondrial processes, including metabolic functions: four solute carrier proteins of the IMM (the tricarboxylate carrier, the calcium-dependent aspartate-glutamate carrier 2, the mitochondrial ornithine transporter 1, and the calcium-binding ATP-Mg/Pi carrier) as well as a small number of respiratory chain complexes I, III, IV, and V subunits (supplemental Table S5), although an overall change in individual complexes was not evident. Several components of the mitochondrial translation machinery were affected across all timepoints, including 13 subunits of the mitochondrial ribosome (supplemental Table S5). Protein quality control and processing proteases including CLPB, matrix localized AAA+ Lon protease, the mitochondrial intermediate peptidase, and the AAA+ chaperone subunit CLPX of the caseinolytic protease complex CLPXP were also collectively altered over the expression time-course (supplemental Table S5). Considering the inner membrane localization of MceC, the effector is in an advantageous position to modulate many of these processes. Interestingly, the voltage-dependent anion channel isoforms 1 and 3 (VDAC1 and 3) and cytochrome *c* were also affected (Fig. 6*B*, supplemental Table S5). The increase in cytochrome *c* levels was also evident via immunoblotting of HEK293^MceC-3XFLAG^ whole cell lysates (supplemental Fig. S4, *A–B*). Mic60 and Mic19 and to a lesser extent, Mic10 and Mic25 proteins of the MICOS complex also displayed increased abundance following MceC expression (Fig. 6*B*). Accordingly, Mic60 levels appeared elevated in immunoblots of HEK293^MceC-3XFLAG^ cell lysates (supplemental Fig. S4, *A*–*B*). This observation was particularly intriguing given Mic19 was also enriched in MceC^3XFLAG^ immunoprecipitation eluates (Fig. 5*B*). These observations using a regulated protein expression system provide a unique snapshot of the impact of MceC localization to the mitochondria in the absence of the full assault of *C. burnetii*-infection and additional effector proteins.

Of the interacting partners enriched within MceC immunoprecipitates, YME1L was particularly interesting. YME1L forms a hexameric, ATP-dependent proteolytic complex in the inner membrane that has emerged as a central regulator of mitochondrial biogenesis, coupling protein quality control with organelle dynamics and function (69). Given the diverse and important roles of YME1L within mitochondria, we decided to further investigate the impact of MceC on YME1L function. When LFQ values of known substrates of YME1L were compared, the vast majority of YME1L substrates underwent little to very subtle changes over the time examined (Fig. 6*C*, supplemental Table S5). Curiously, four YME1L substrates were significantly altered with a decreasing trend across all time points (Fig. 6*C* and supplemental Table S5). These were the inner membrane localized nucleoside diphosphate kinase NME4, the PIP3-binding outer membrane protein FUNDC2, CLPB, and the Coenzyme A degrading enzyme NUDT19. Grouping of these substrate proteins according to their mitochondrial function did not reveal any collective changes to a mitochondrial process; however, it is interesting to note that in addition to being significantly altered across all time-points, NME4 was attributed to lipid transport. Thus, the interaction of MceC with YME1L does not appear to lead to a broad change in the proteolytic capacity of YME1L and may instead be a more targeted regulation of a subset of proteins important to *C. burnetii* infection.

## Discussion

The development of techniques to endogenously study host–pathogen interactions is integral for the progression of the field. Adaptation of high-sensitivity technology such as super resolution microscopy, mass spectrometry, and electron cryotomography to detect and determine the structure of a protein or protein complexes present exciting opportunities to advance the toolkit available to understand how pathogens manipulate the host cell during infection. In this study, we develop a proteomics-based screening approach to identify *C. burnetii* effector proteins targeted to distinct subcellular compartments and demonstrate the application of this technique to identify mitochondrially-targeted effector proteins. Mass spectrometry presented an elegant tool to achieve this as high-sensitivity mass spectrometers allows identification of proteins present in relatively low abundance, such as an effector protein amidst the host cell proteome (70). This is the first study to utilize a broader, unbiased, and unlabeled approach to capture the interactions of native *C. burnetii* proteins with the host cell mitochondrion. Development of these methodologies is particularly important for pathogens with large effector repertoires where a key outstanding question remains: are these proteins *bona fide* effectors that are translocated into the host cell during infection (71)?

This nontargeted approach facilitated identification of seven proteins associated with the mitochondria during natural infection. Of the seven *C. burnetii* proteins associated with the organelle during infection, five have been shown to be translocated by the T4SS into the host cell (24, 72). Of the known effector proteins further investigated for mitochondrial localization, CBU0937, CBU1425, CBU1594, and CBU1677 displayed association with the mitochondria validating their proteomic enrichment with the organelle. While, we focused our analysis on macrophage-like cells, it is possible that the cohort of mitochondrial-targeted *C. burnetii* effector proteins could differ between other cell types. Curiously, transposon mutagenesis screening has demonstrated that CBU0937 makes a significant contribution to the intracellular replication of *C. burnetii*, further implicating the importance of the host cell mitochondrion during infection (64). Further characterization of CBU1425 revealed that the protein is imported and integrated into the IMM and may influence organelle quality control systems. Thus, we propose the renaming of these four effector proteins to Mitochondrial *Coxiella* effector protein B–E (MceB–E). These proteins join the previously identified mitochondrial-localized effector MceA, forming a cohort of *C. burnetii* proteins that target the organelle during infection.

The techniques and analytical approaches developed here provide an exciting platform that could be applied to the study of other organelles during *C. burnetii* infection, and more broadly, to the study of other intracellular bacterial pathogens. Previous studies have proteomically examined bacterial containing vacuoles, but few have attempted to characterize host organelles during infection (73, 74). Expanding this study spatially and temporally would provide a systematic, comprehensive analysis of effector protein localization and dynamics within the host cell over the full course of *C. burnetii* infection. If or when bacterial effector proteins traffic between multiple organelles during infection is not well understood. Investigations into the function of *C. burnetii* effector AnkG demonstrated the protein is capable of trafficking between the mitochondria and the nucleus via a “piggy-backing” mechanism to interrupt nuclear apoptotic signaling (38, 75). Our analysis did not capture enrichment of AnkG at the mitochondrion; however, it is possible that this interorganelle trafficking had already resulted in translocation of the protein to the nucleus. Considering *C. burnetii* infection persists in the host cell for approximately 7 days, it is possible that effector proteins could be dually localized or localized to membrane contact sites between organelles at distinct stages of infection. Spatial and temporal application of the techniques developed here could form a “road map” of effector targeting and trafficking events, assisting in functionally distinguishing between effector proteins, a particular advantage in bacterial species with large effector protein repertoires such as *C. burnetii*.

Introduction of bacterial proteins into the host cell by the T4SS can impact how an effector protein behaves within the cell. Structural characterization of the *L. pneumophila* T4SS has provided valuable insights into deciphering the translocation mechanism underlying this complex (8, 9). What is clear from these studies is that delivery of a native, T4SS-translocated effector protein is distinct to protein targeting through the ER or secretory pathway as may occur during ectopic expression. This was evidenced previously during our characterization of MceA (28). Examination of our identified effector proteins with both N- and C-terminal FLAG tags in both uninfected and infected cells revealed interesting aspects of effector protein biogenesis. For instance, MceE (CBU1677) demonstrated distinct localization only when expressed in infected cells, indicating a potential role for additional *C. burnetii* proteins in aiding this targeting. Mitochondrial targeting of MceB (CBU0937) and MceC was observed only when the Nterminus of the protein was exposed, suggesting the mitochondrial targeting information for these effector proteins is located in this region. Bacterial effector proteins frequently hijack host targeting signals to direct the protein to specific cellular locations (32, 76). In the case of the mitochondrion, the evolutionary relationship between the organelle and Gram-negative bacteria could be exploited to aid in effector protein targeting. Several lines of evidence indicate that bacterial proteins are capable of utilizing the mitochondrial import machinery for entry into the organelle (77, 78, 79, 80, 81, 82, 83). Bioinformatic analysis of the domain structure of MceB predicts the protein contains β-sheet topology and homology to bacterial porin proteins. β-barrel proteins of *Neisseria* sp. PorB and Omp85 have been shown to successfully utilize the mitochondrial import machinery for import and assembly into mitochondrial membranes (80, 81, 84). PorB association with the host cell mitochondria disrupts the membrane potential, sensitizing cells to apoptosis (80). Sequence analysis indicates that MceB lacks the classical β-targeting signal of mitochondrial β-barrel proteins (85). Considering the homology it has to porin-like proteins, it will be interesting to determine the precise interaction of this effector protein with human mitochondria during *C. burnetii* infection.

Before this study, MceC was a functionally uncharacterized *C. burnetii* effector protein (24). Mitochondrial subfractionation and carbonate extraction revealed the protein is imported into the organelle and integrated into the IMM, with the C-terminus of the protein in the IMS. MceC does not contain a classical mitochondrial targeting sequence, suggesting it is not a TIM23 substrate. Curiously, the substrate spectrum of human TIM22 complex recently expanded to include non-canonical substrates containing two or three transmembrane segments (86). It is interesting to postulate that *C. burnetii* may exploit a non-canonical route to import MceC into mitochondria. Immunoprecipitation of MceC identified several, resident mitochondrial putative interacting partners, including components of the mitochondrial quality control machinery, namely the i-AAA protease, YME1L, and CLPB. Proteins of the mitochondrial quality control network include chaperones and proteolytic enzymes that survey and maintain mitochondrial homeostasis in response to organelle and cellular stresses (87, 88). Although in our system, MceC was expressed at a low level, we cannot rule out that these interactors are responding to the presence of MceC and attempting to remove the protein to maintain organelle proteostasis. Whether these interactions are direct remains to be defined, but MceC also immunoprecipitates additional components of the YME1L quality control network including SLP2, a component of the SLP2-YME1L-PARL (SPY) proteolytic complex and OPA1 (89). Notably, immunoprecipitation of MceC in the context of *C. burnetii* infection revealed conservation of the interaction with SLP2 but not YME1L. These observations suggest interaction of MceC with these mitochondrial proteins represents a functional interaction; however, this needs to be experimentally investigated further. Recently, YME1L substrates were identified using quantitative proteomics in Yme1l^−/−^ MEF cells (90). Using proteomics analysis coupled to a time-course of MceC expression, we were able to rule out the collective modulation of a functional subset of YME1L substrates. Understanding the importance of MceB–E to *C. burnetii* infection by the characterization of *C. burnetii* mutants lacking these genes could provide further clues to the proteins function during infection and in response to various cellular stresses. Conversely, analysis of *C. burnetii* replication in human cell lines lacking MceC interacting partners could also shed light on the significance of mitochondrial processes during *C. burnetii* infection.

This research has showcased the power of unbiased proteomics-based techniques to investigate and understand the host–pathogen interactions occurring between the host cell mitochondrion and the intracellular bacterial pathogen *C. burnetii*. This has established a method to investigate effector protein targeting during native infection and revealed four additional, mitochondrially-targeted *C. burnetii* effector proteins. The techniques developed here demonstrate the feasibility of applying mass spectrometry to create a map of the subcellular dynamics of native effector proteins during intracellular bacterial infections.

## Data availability

The mass spectrometry proteomics data have been deposited to the ProteomeXchange Consortium via the PRIDE (91, 92) partner repository with the dataset identifier PXD019322 (mitochondrial purification from *C. burnetii*-infected cells), PXD019417 (MceC affinity purification and interactome analysis) and PXD021704 (Quantitative mitochondrial proteome analysis during MceC expression time-course).

## Conflict of interest

The authors declare no competing interest.
