# Supplementary material for: Proteomic Identification of Coxiella burnetii Effector Proteins Targeted to the Host Cell Mitochondria During Infection
Source: Mol Cell Proteomics. 2020 Dec 3;20:100005. doi: 10.1074/mcp.RA120.002370 (PMC7950127; doi:10.1074/mcp.RA120.002370)

Gene Name: tolR CBU_1575

Raw File	Scan	Method	Score	m/z
180615_LauraF_Cr1	33591	FTMS; HCD	48.41	482.76

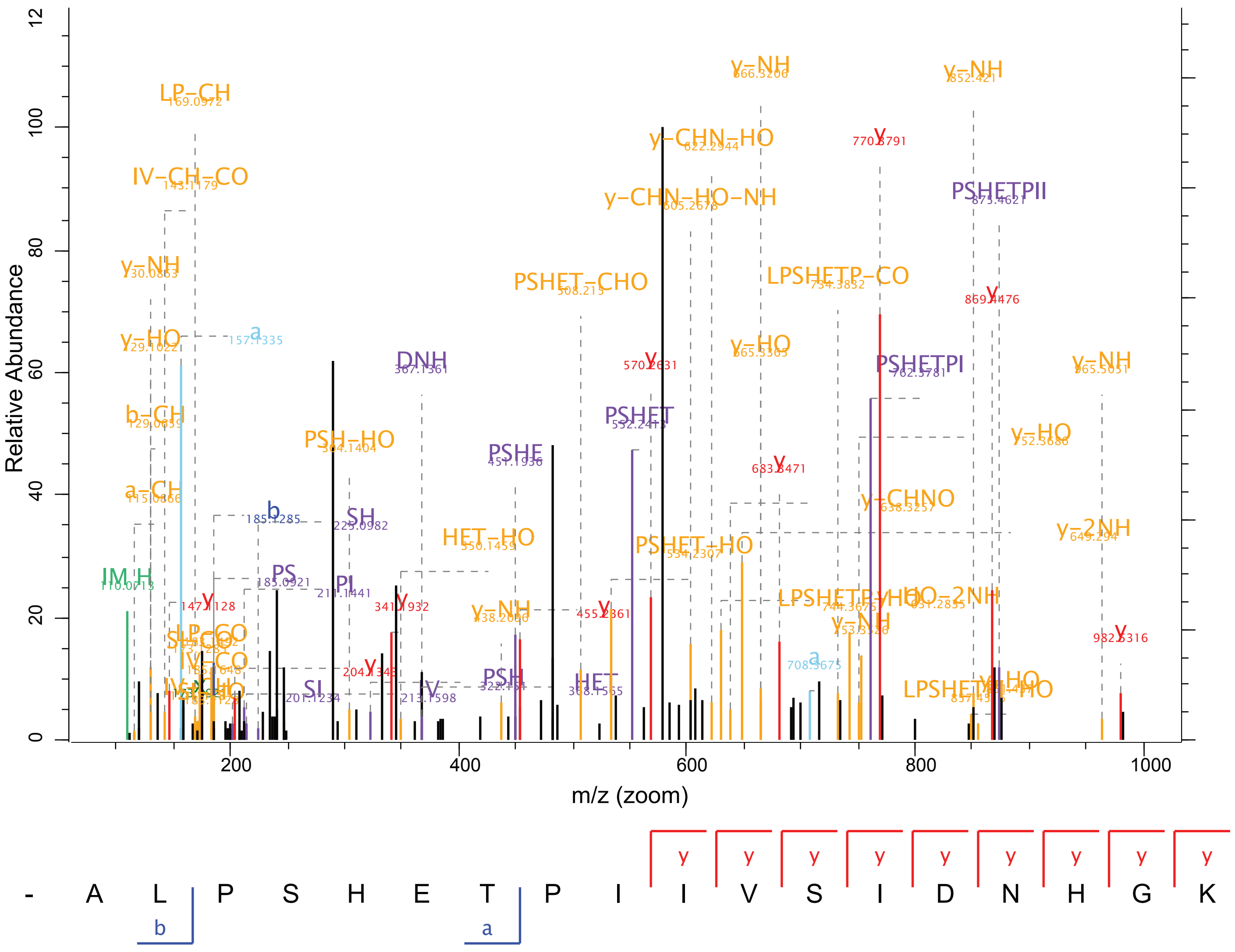

-

Gene Name: CNIH4

Raw File	Scan	Method	Score	m/z	Gene names
180615_LauraF_Cr1	72117	FTMS; HCD	45.7	760.03	CNIH4

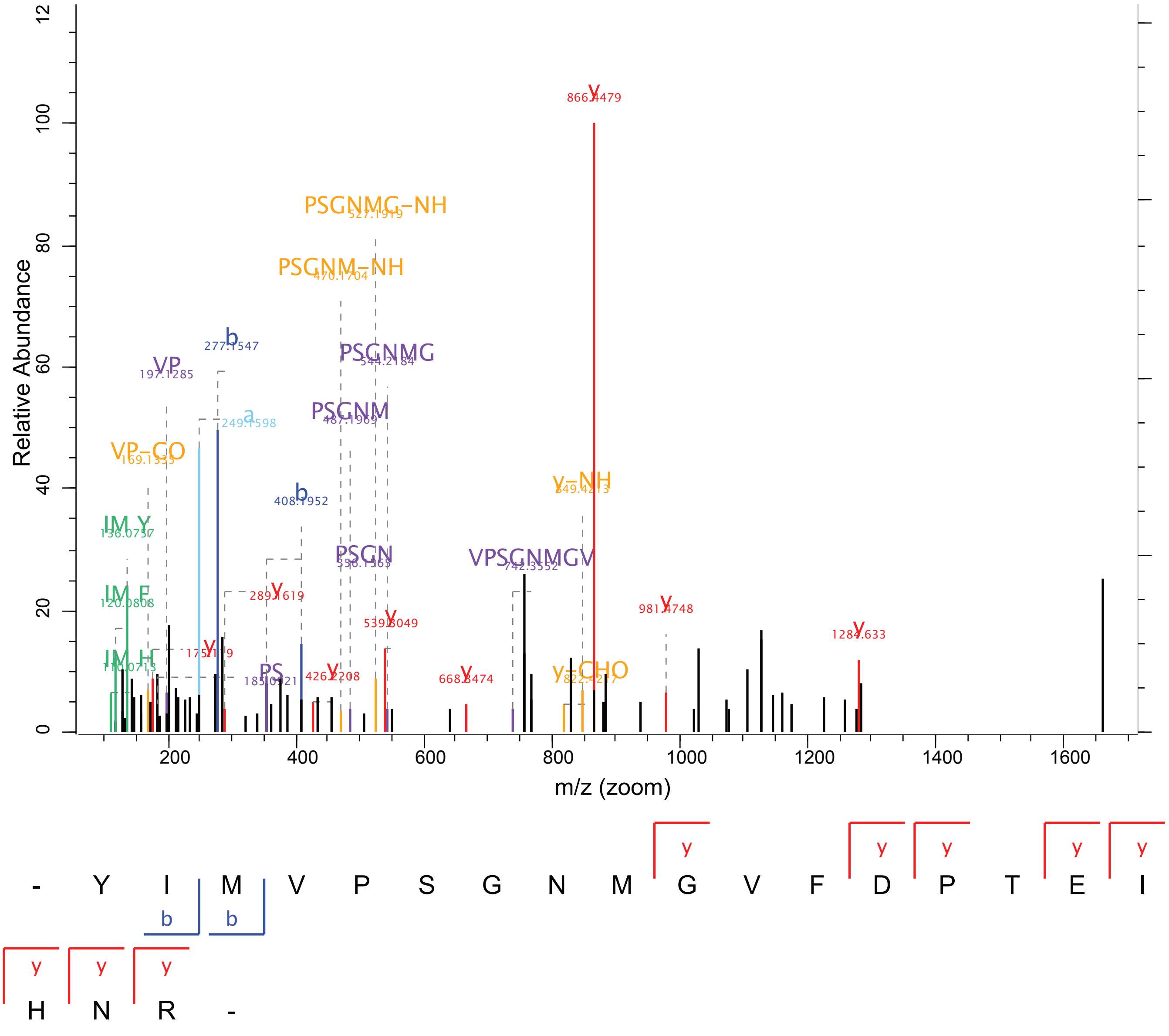

Gene Name: CBU_0584

Raw File	Scan	Method	Score	m/z
180615_LauraF_Cr1	13258	FTMS; HCD	81.32	630.62

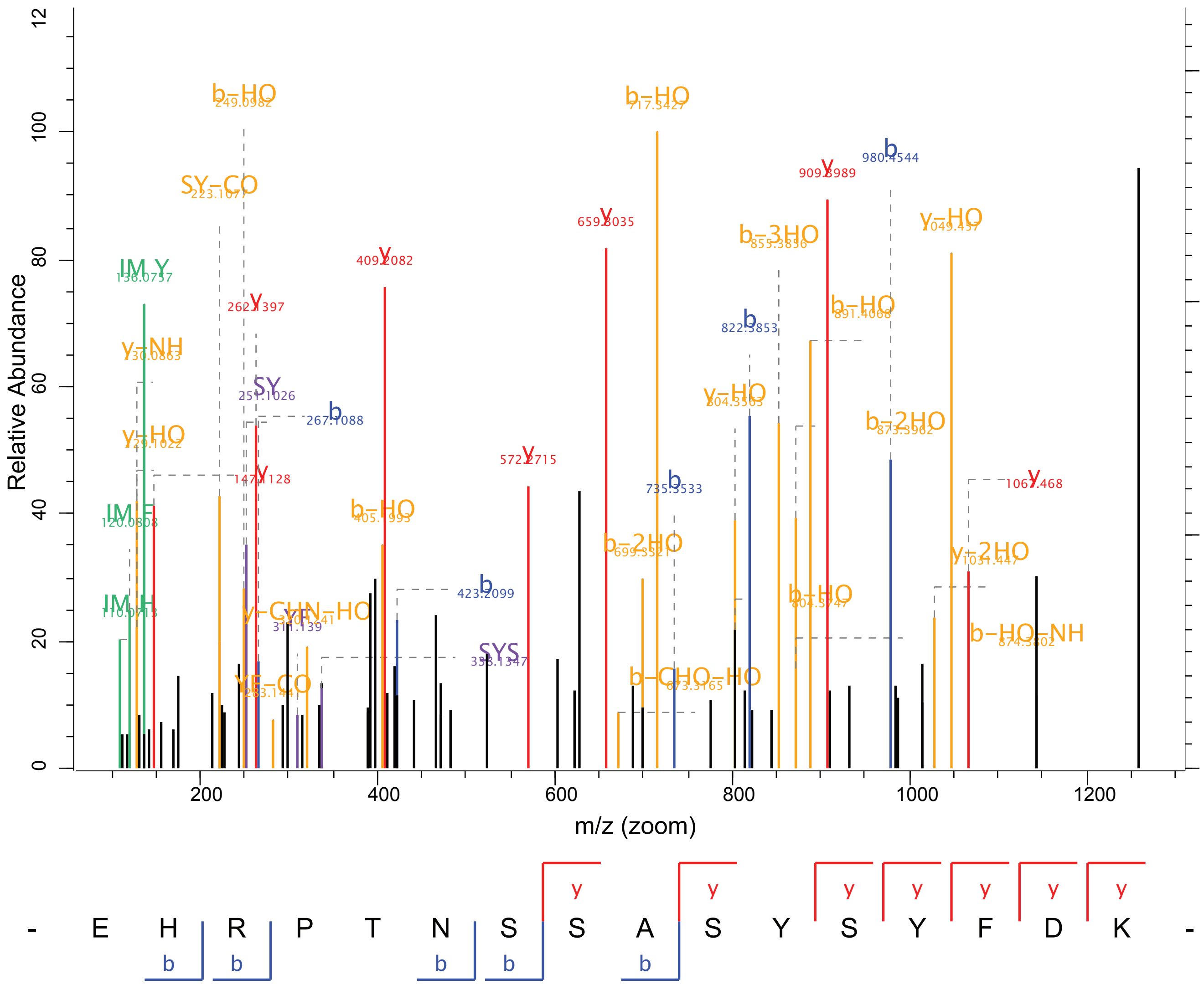

Fielden et al., 2020_Supporting Data_Supplementary Table 1

Gene Name: ATP5G1;ATP5G2;ATP5G3

Raw File	Scan	Method	Score	m/z	Gene names
180615_LauraF_CR2	115652	FTMS; HCD	81.72	1405.74	ATP5G1;ATP5G2;ATP5G3

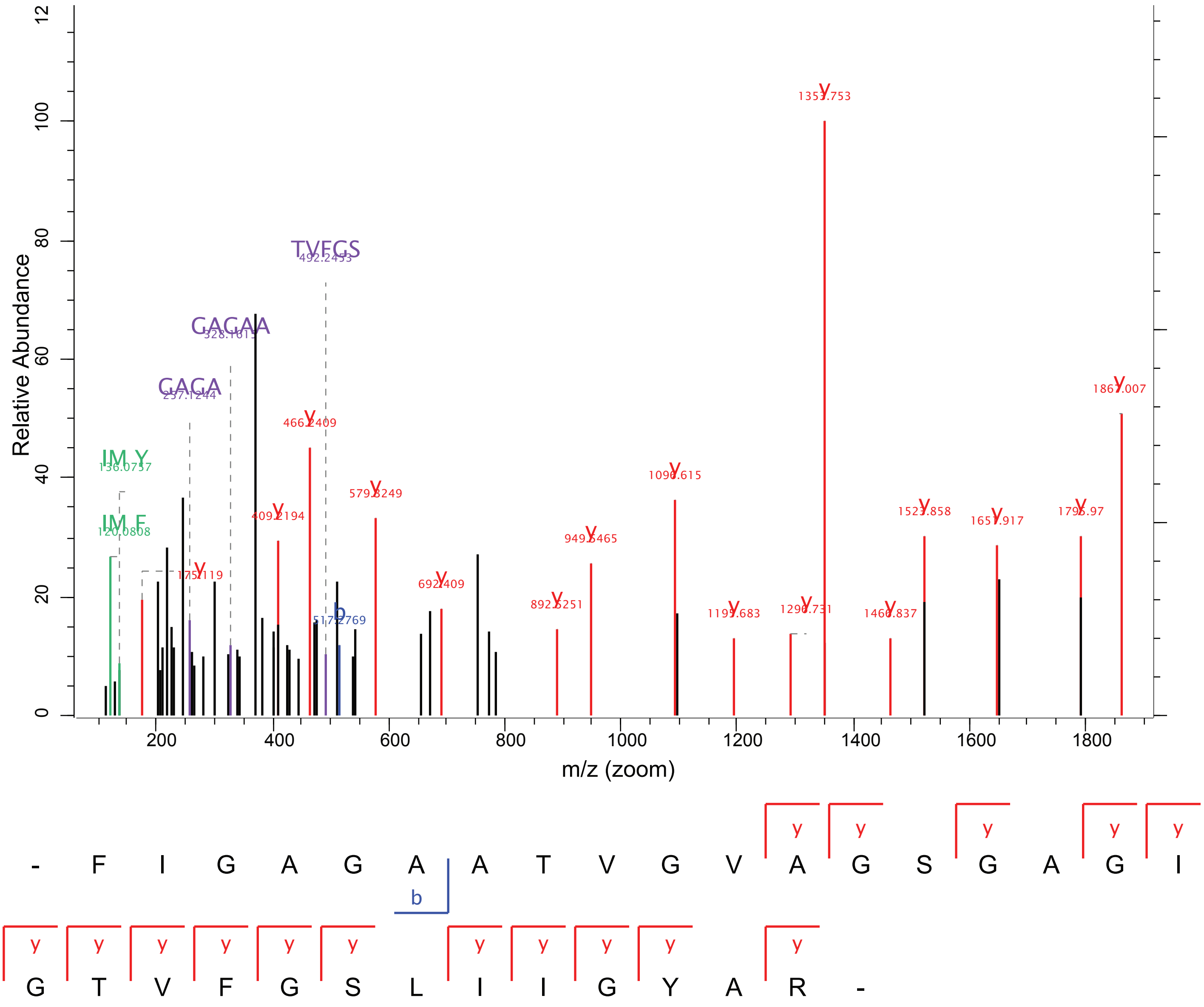

Fielden et al., 2020_Supporting Data_Supplementary Table 1

Gene Name: TMEM41B

Raw File	Scan	Method	Score	m/z	Gene names
180615_LauraF_PR2	5631	FTMS; HCD	149.32	916.95	TMEM41B

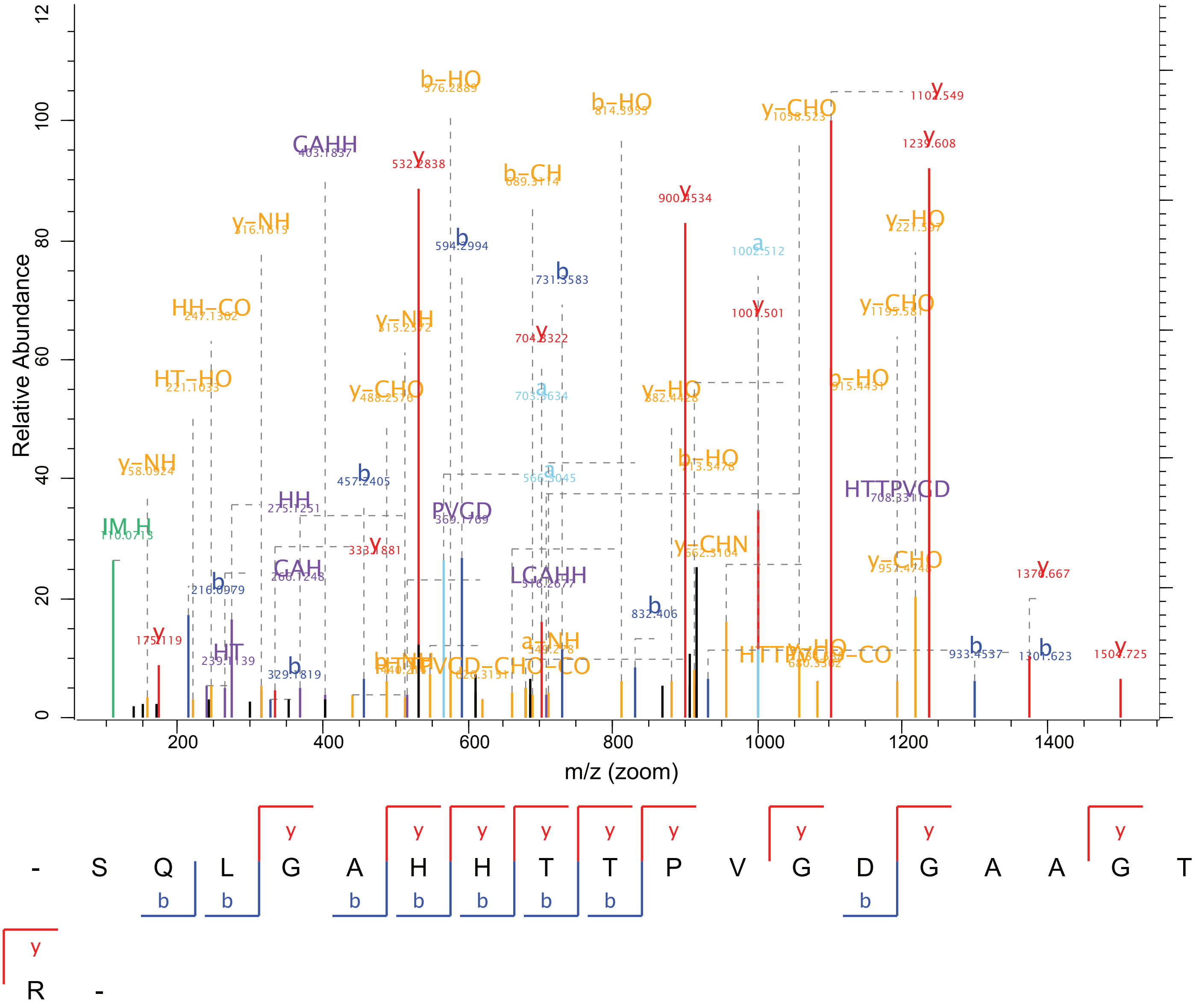

Gene Name: TMEM258

Raw File	Scan	Method	Score	m/z	Gene names
180615_LauraF_PR1	24147	FTMS; HCD	152.47	483.72	TMEM258

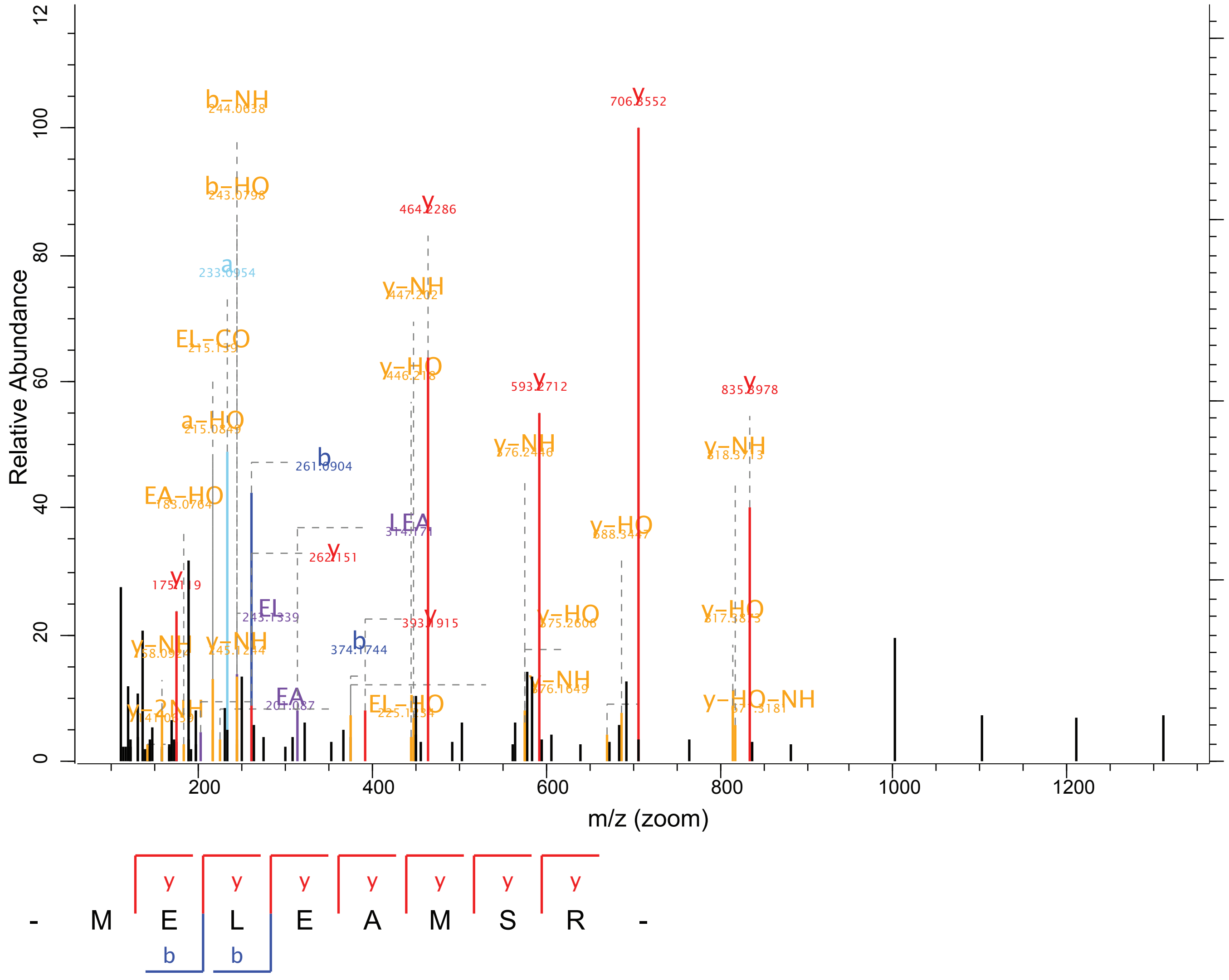

Fielden et al., 2020_Supporting Data_Supplementary Table 1

Gene Name: RPL39P5;RPL39

Raw File	Scan	Method	Score	m/z	Gene names
180615_LauraF_PR3	20867	FTMS; HCD	105.1	436.92	RPL39P5;RPL39

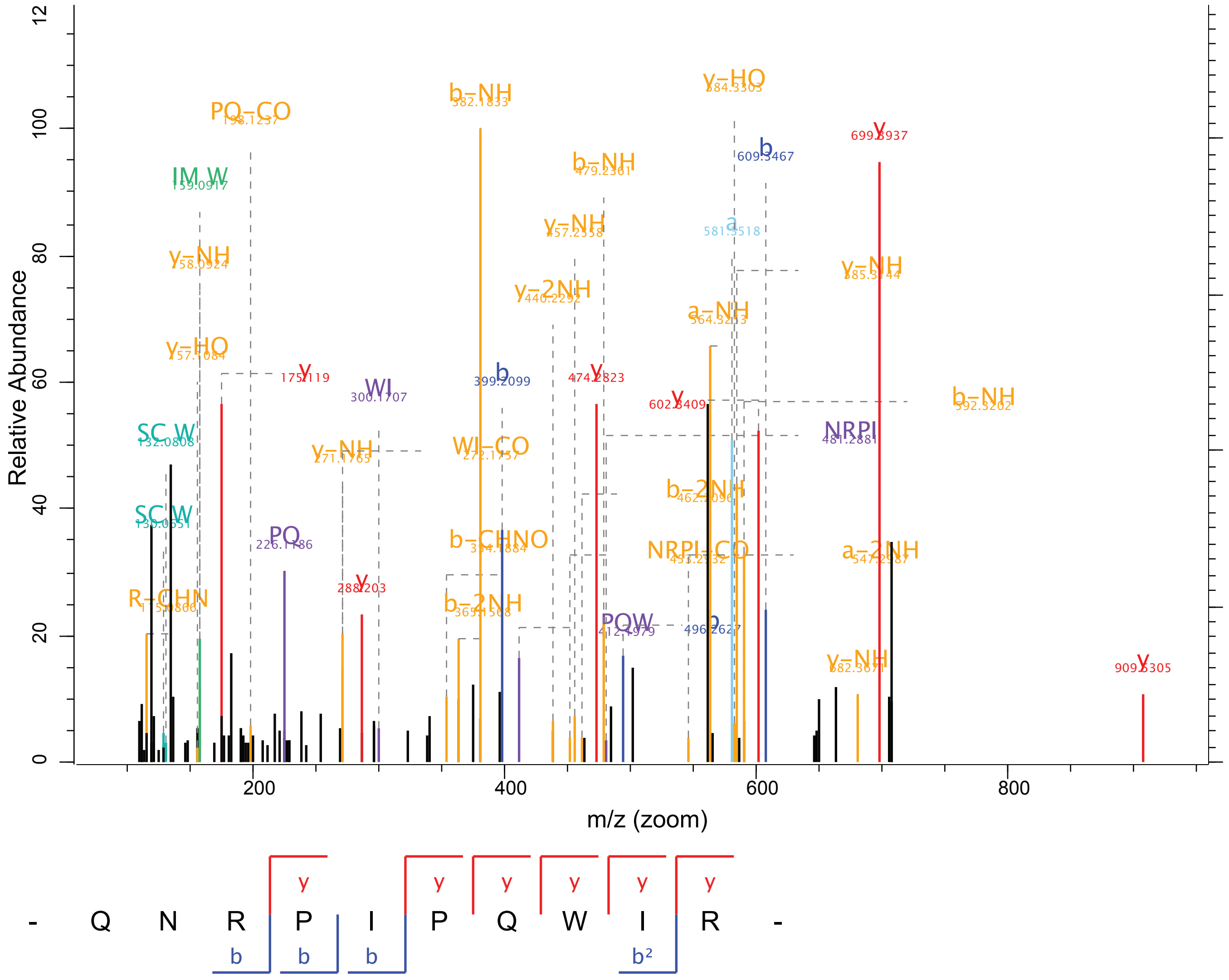

Gene Name: RP13-360B22.2;TMEM164

Raw File	Scan	Method	Score	m/z	Gene names
180615_LauraF_PR3	5489	FTMS; HCD	69.45	955.96	RP13-360B22.2;TMEM164

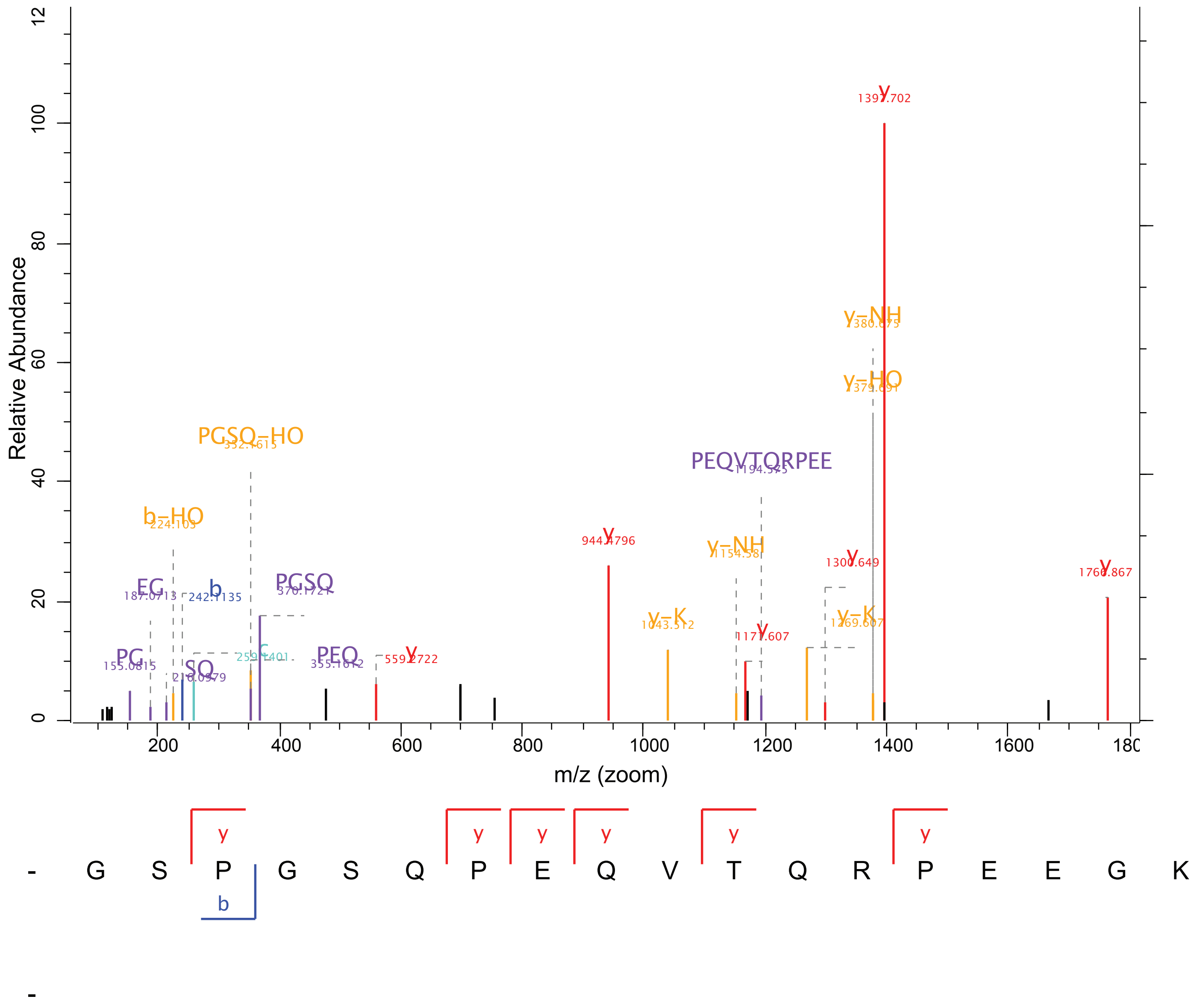

Gene Name: C20orf24

Raw File	Scan	Method	Score	m/z	Gene names
180615_LauraF_CR3	10142	FTMS; HCD	162.22	888.49	C20orf24

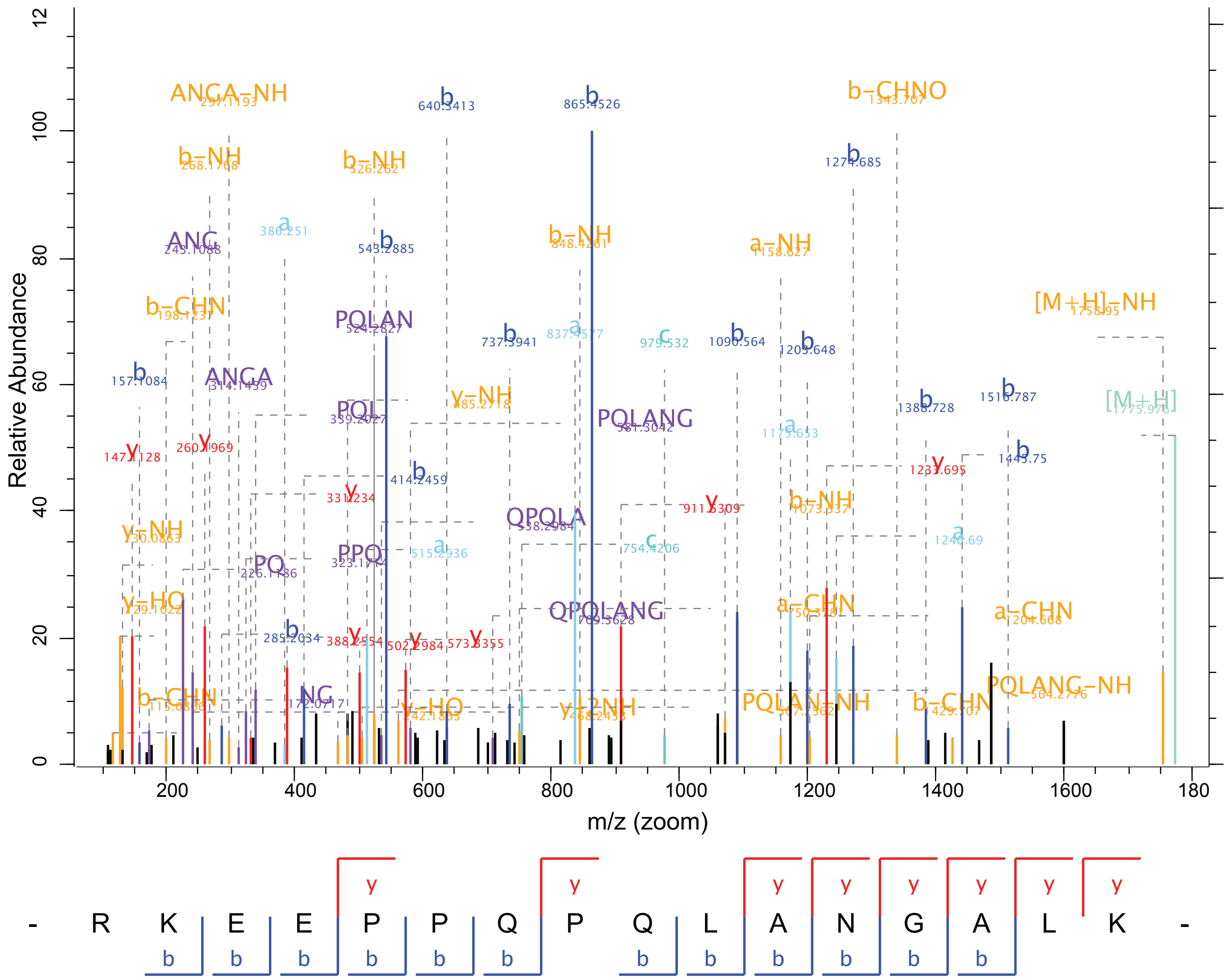

Supplement: Supporting_Data_1 [file mmc6.pdf]
