# Supplementary material for: Proteomic Identification of Coxiella burnetii Effector Proteins Targeted to the Host Cell Mitochondria During Infection
Source: Mol Cell Proteomics. 2020 Dec 3;20:100005. doi: 10.1074/mcp.RA120.002370 (PMC7950127; doi:10.1074/mcp.RA120.002370)

Gene Name: SDHD

Raw File	Scan	Method	Score	m/z	Gene names
200320_Laura_LF_496_13	14238	FTMS; HCD	150.17	579.29	SDHD

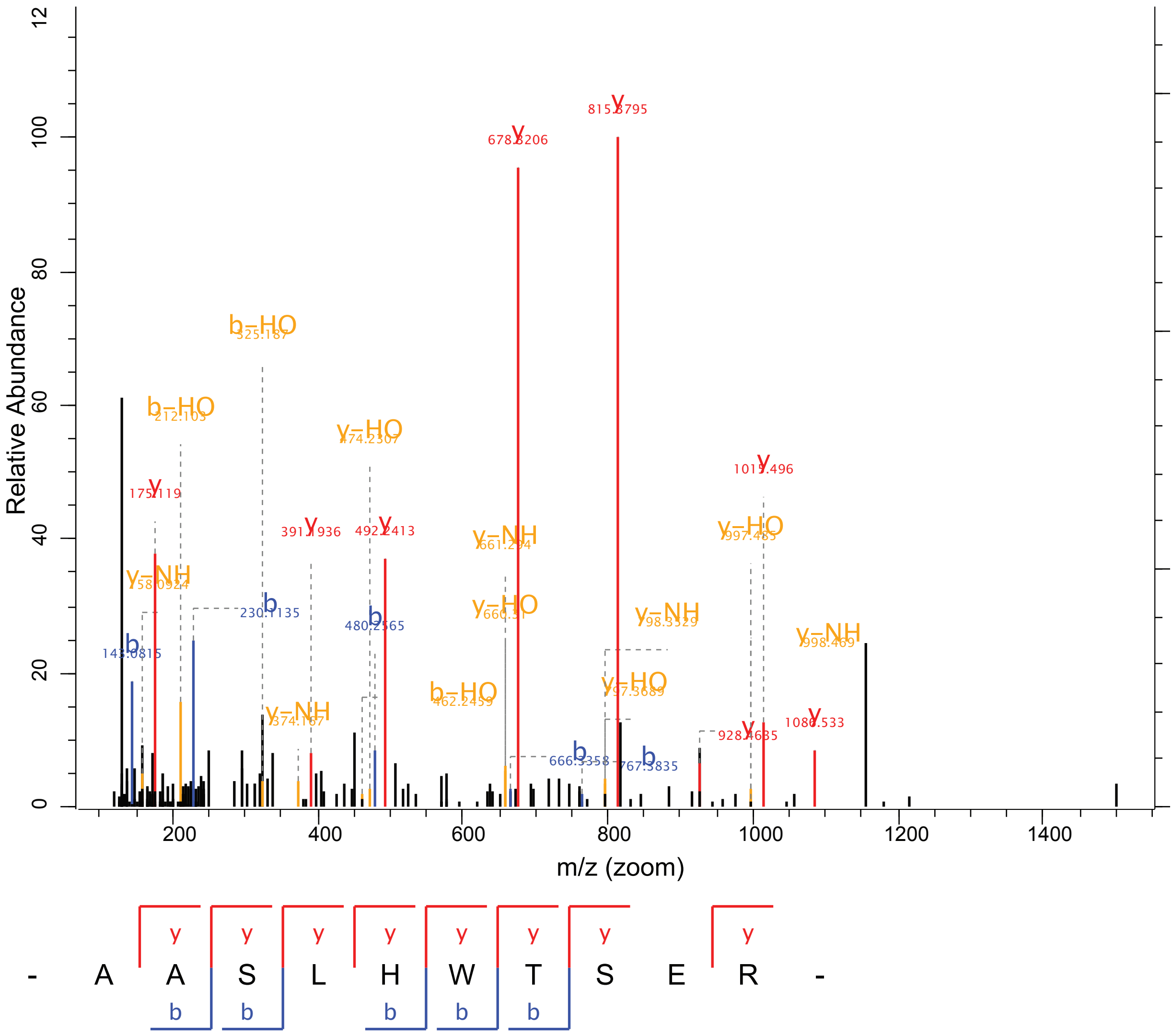

Gene Name: MINOS1

Raw File	Scan	Method	Score	m/z	Gene names
200320_Laura_LF_496_14	17649	FTMS; HCD	207.88	702.85	MINOS1

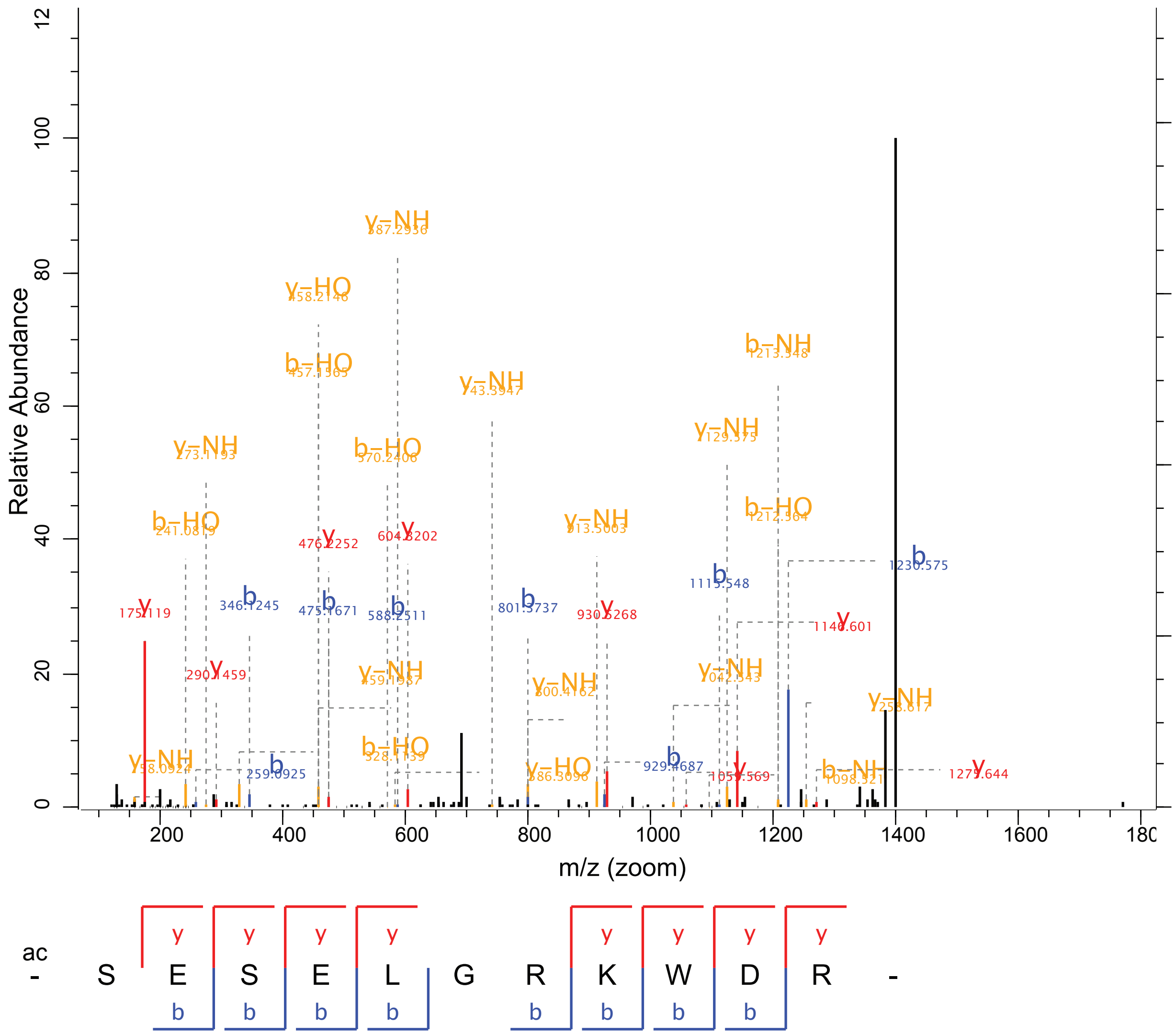

Gene Name: NDUFB2

Raw File	Scan	Method	Score	m/z	Gene names
200320_Laura_LF_496_15	21074	FTMS; HCD	282.57	604.83	NDUFB2

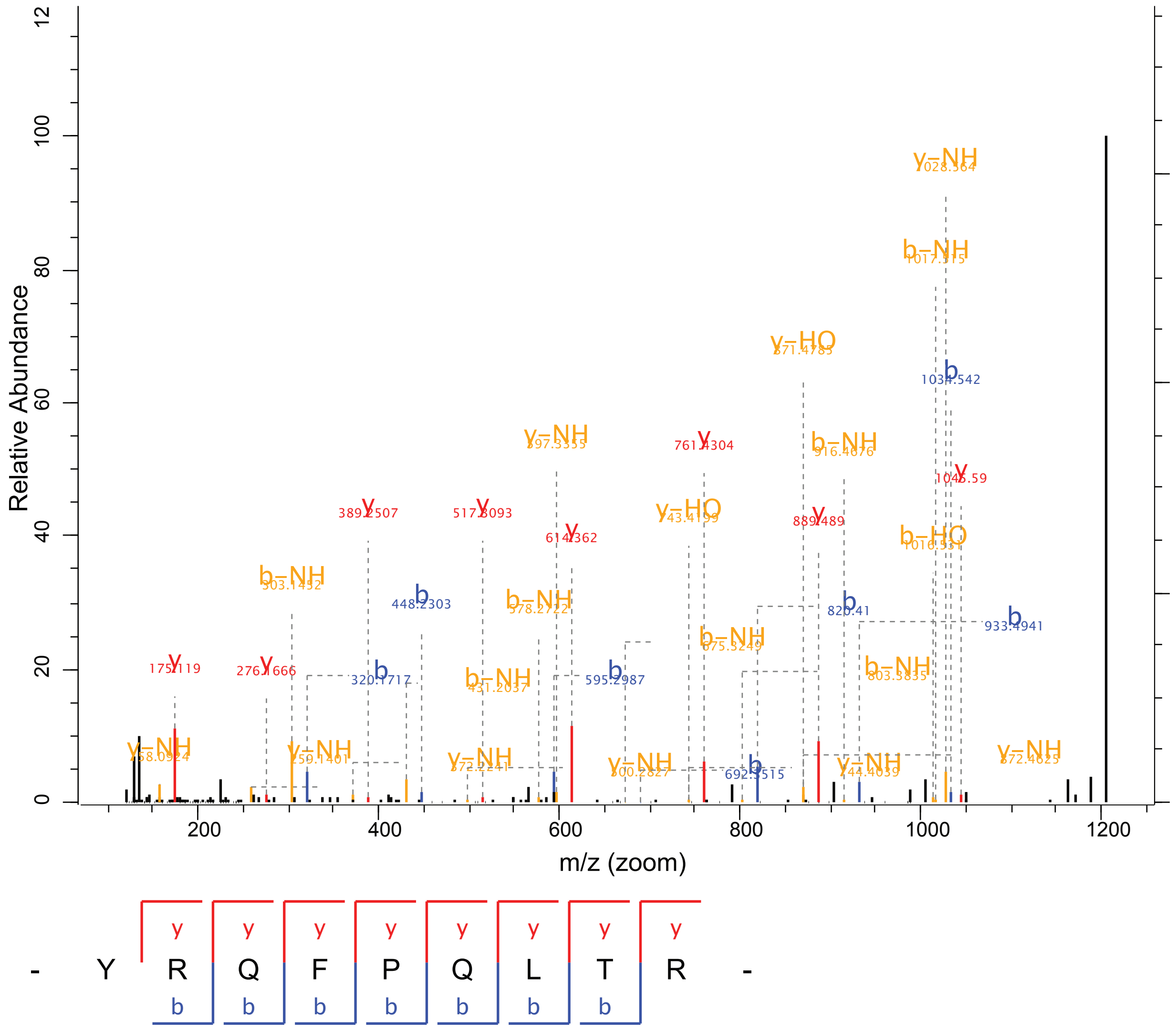

Gene Name: PNKD

Raw File	Scan	Method	Score	m/z	Gene names
200320_Laura_LF_496_6	40303	FTMS; HCD	146.34	950.47	PNKD

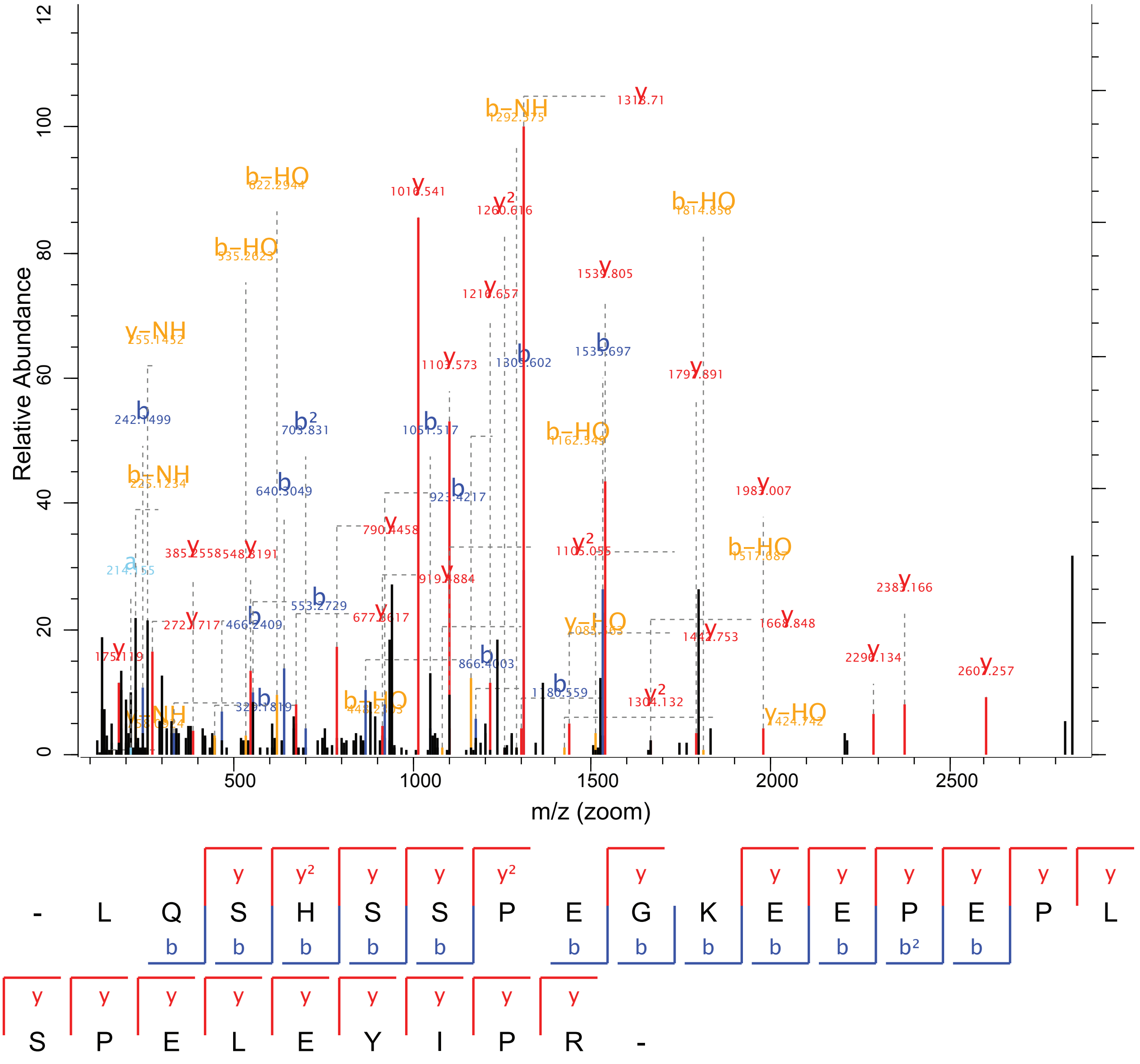

Gene Name: MRPS18C

Raw File	Scan	Method	Score	m/z	Gene names
200320_Laura_LF_496_12	47024	FTMS; HCD	53.34	805.41	MRPS18C

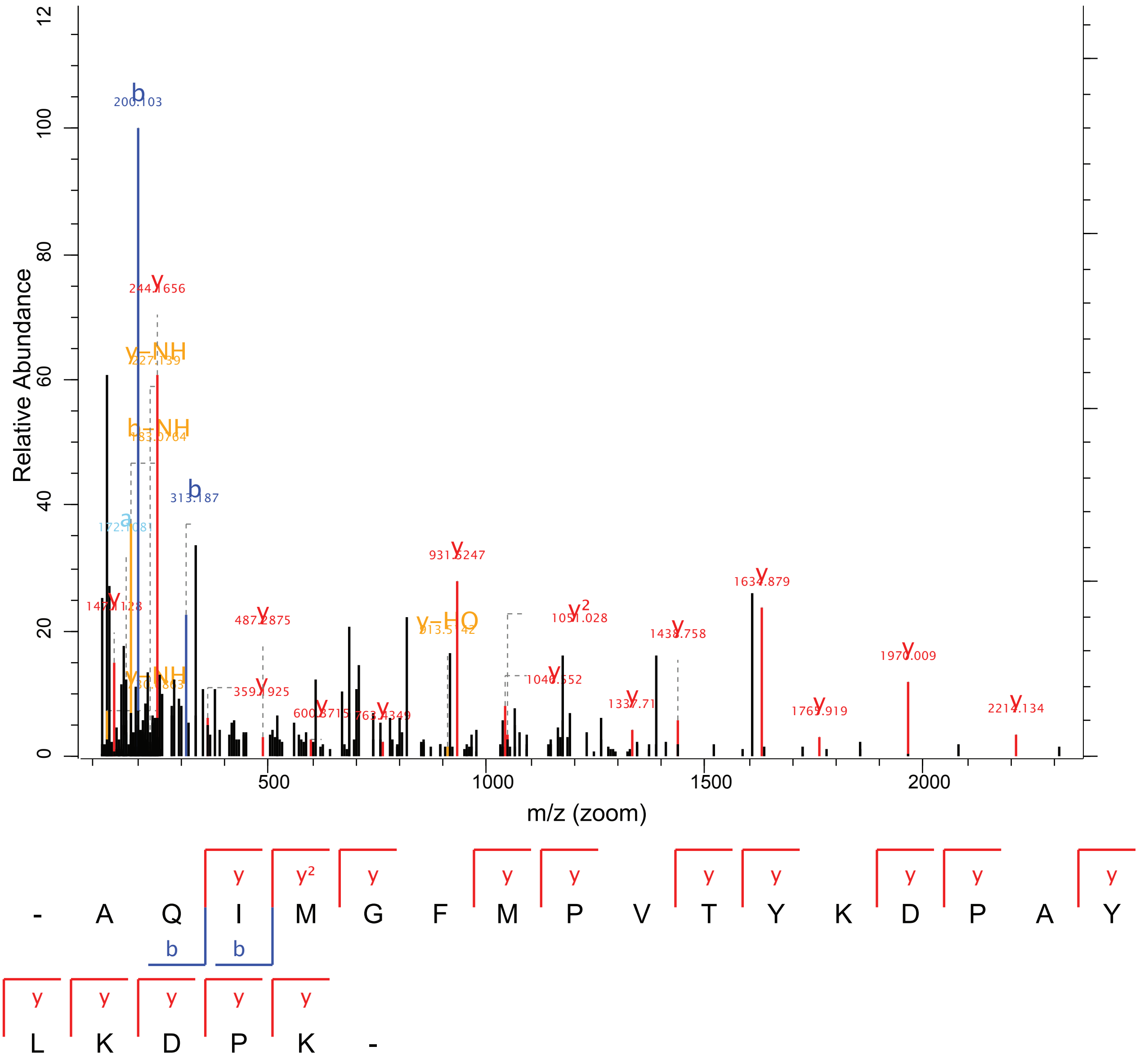

Supplement: Supporting_Data_2 [file mmc7.pdf]
