# Supplementary material for: Proteomic Identification of Coxiella burnetii Effector Proteins Targeted to the Host Cell Mitochondria During Infection
Source: Mol Cell Proteomics. 2020 Dec 3;20:100005. doi: 10.1074/mcp.RA120.002370 (PMC7950127; doi:10.1074/mcp.RA120.002370)
Supplement: Supplemental Figures [file mmc8.pdf]

Proteomic identification of *Coxiella burnetii* effector proteins targeted to the host cell mitochondria during infection.

Laura F. Fielden[1], Nichollas E. Scott[2], Catherine S. Palmer[1], Chen Ai Khoo[2], Hayley J Newton[2,**] and Diana Stojanovski[1,**]

Supplementary Figures and Figure Legends

Supplementary Figure Legends

Supplementary Figure 1: Comparison of mitochondrial network morphology during *C. burnetii* infection. HeLa and differentiated THP-1 macrophage-like cells were left uninfected or infected with *C. burnetii.* Cells were fixed and immunodecorated for mitochondrial Tom20 (red) and DNA stained with Hoechst (blue). Scale bar represents 10 µm. Asterix represents location of *Coxiella*-containing vacuole (CCV).

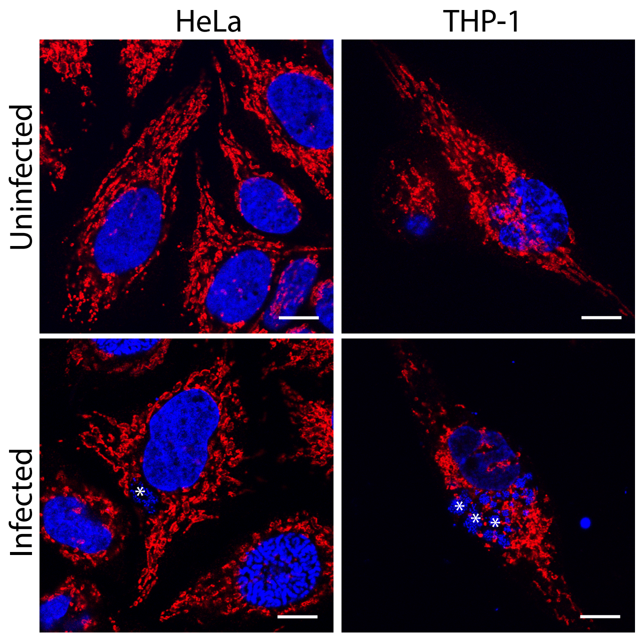

Fielden et al., 2020_Supplementary Figure 1

Supplementary Figure 2: Solubility of MceC^{3XFLAG}. (**A**) Schematic representation of predicted domain structure of MceC. Location of predicted transmembrane domains (pink), glycine motifs (red) and region of homology to 'surface antigen domain'-containing proteins (blue) depicted. Numbers denote amino acid locations. (**B** and **C**) Mitochondria were isolated from HEK293$^{MceC\text{-}3XFLAG}$ cells induced with tetracycline (1 μg/mL) for (B) 4 HRS or (C) 8 HRS, were solubilised in digitonin (1% (w/v)), TX-100 (1% (v/v)) or DDM (0.2% and 0.4% (w/v)). Solubilised samples were separated by centrifugation and the total (T), pellet (P) and supernatant (SN) fractions analysed by SDS-PAGE and immunoblotting with the indicated antibodies. (**D)** HEK293$^{MceC\text{-}3XFLAG}$ cells were treated for 16 hours with tetracycline (1 μg/mL) before being fixed and immunodecorated with antibodies against FLAG (green) and mitochondrial outer membrane protein Tom20 or mitochondrial inner membrane protein Tim50 (red). Nucleus was stained with Hoechst (blue). Scale bar represents 10 μm. Dashed box represents location of zoom image in ***Figure 4E***. (**E**) Proposed localisation of MceC within the mitochondria.

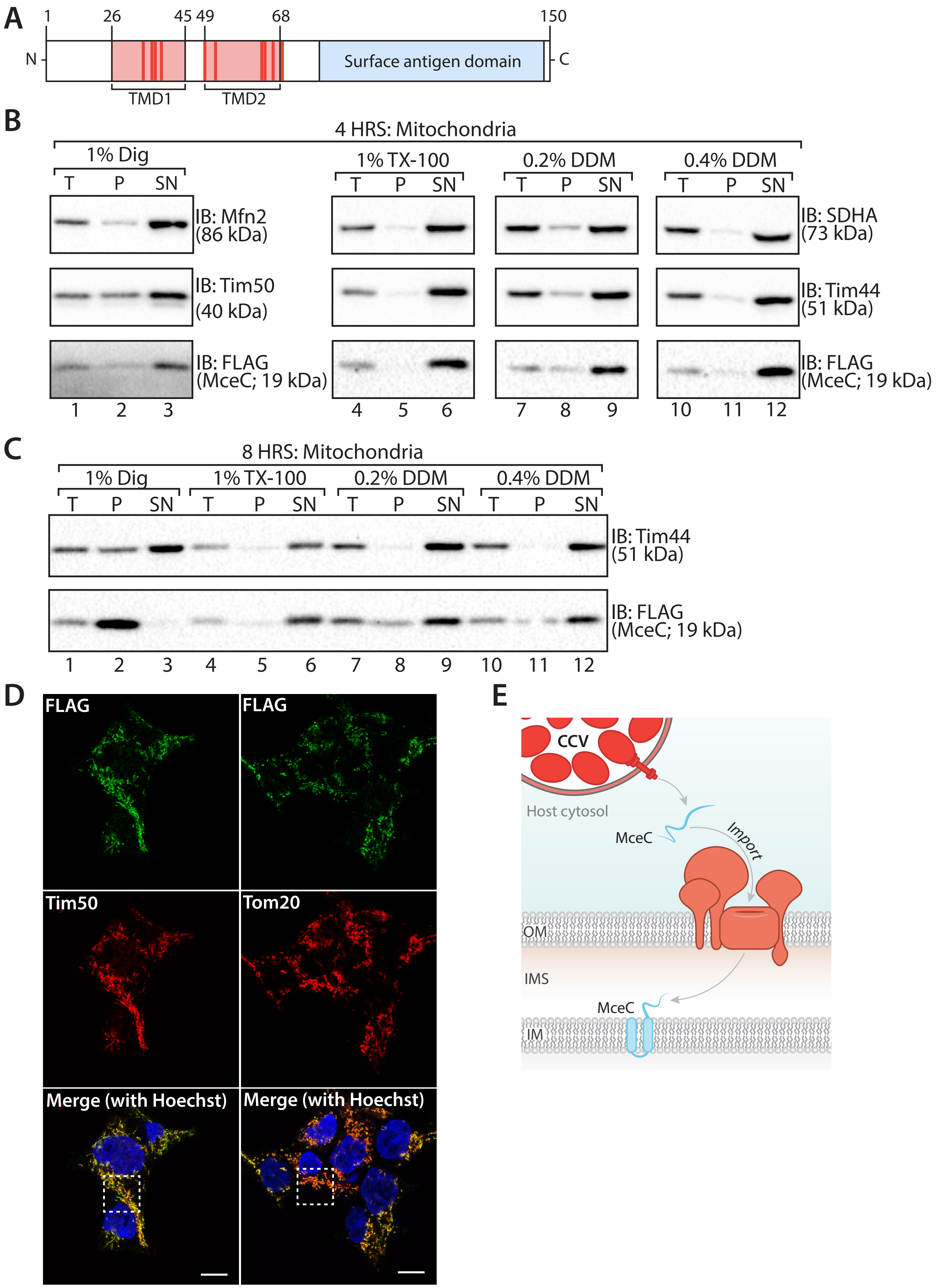

Fielden et al., 2020_Supplementary Figure 2

Supplementary Figure 3: MceC immunoprecipitates resident mitochondrial proteins during *C. burnetii* infection. Mitochondria isolated from *C. burnetii*-infected control and HEK293 cells expressing $MceC^{3XFLAG}$ for 4-hours were solubilised in digitonin (1% (w/v))-containing buffer and lysates subjected to anti-FLAG immunoprecipitation. Volcano plot showing proteins enriched following MceC immunoprecipitation compared to control 'empty vector' (EV) cells. Mitochondrial proteins plotted and each circle represents one protein. Gene names of selected proteins are used for labels. Horizontal axis shows the Log_2(fold change) of MceC interacting partners and vertical axis shows -Log_{10}(p-value) of two-sample Students T-test (FDR: 0.01 and s0: 0.1). n = 3 biological replicates. QC: Quality Control, IM: inner membrane. Colours correspond to key.

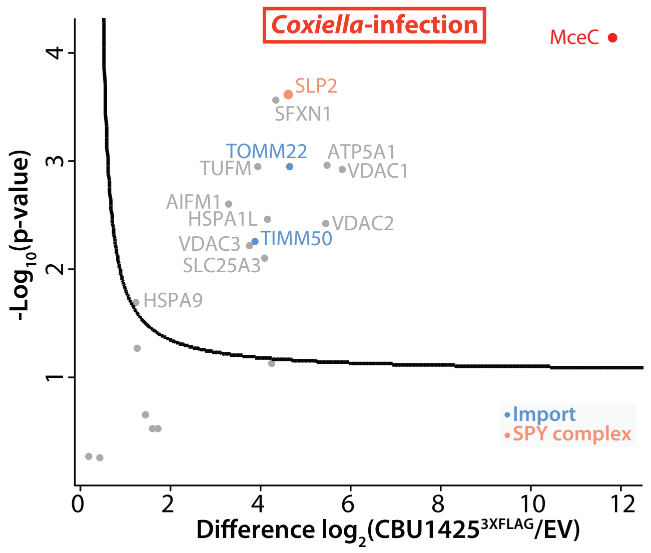

Fielden et al., 2020_Supplemental Figure 3

Supplementary Figure 4: (**A**) Whole cell extracts from HEK293 control (expressing an empty vector (EV)) or $MceC^{3XFLAG}$ cells induced with tetracycline for the indicated times were separated by SDS-PAGE and analysed by immunoblotting with the indicated antibodies. (**B**) Quantification of immunoblots in (A). Relative protein levels of cytochrome *c*, VDAC1, Tim29 and Mic60 were quantified and presented as mean ± SEM, across minimum 2 independent biological replicates. Quantitation was performed using BioRad Image Lab quantitation software. The band intensity of the indicated antibodies was compared to that of the control (actin) to determine the relative level for each protein at each timepoint. Relative protein levels across conditions (control (EV) and MceC) were determined by dividing the relative level of each protein following MceC expression by the relative level in the control (EV). Values for each time point were converted to percentage and graphed.

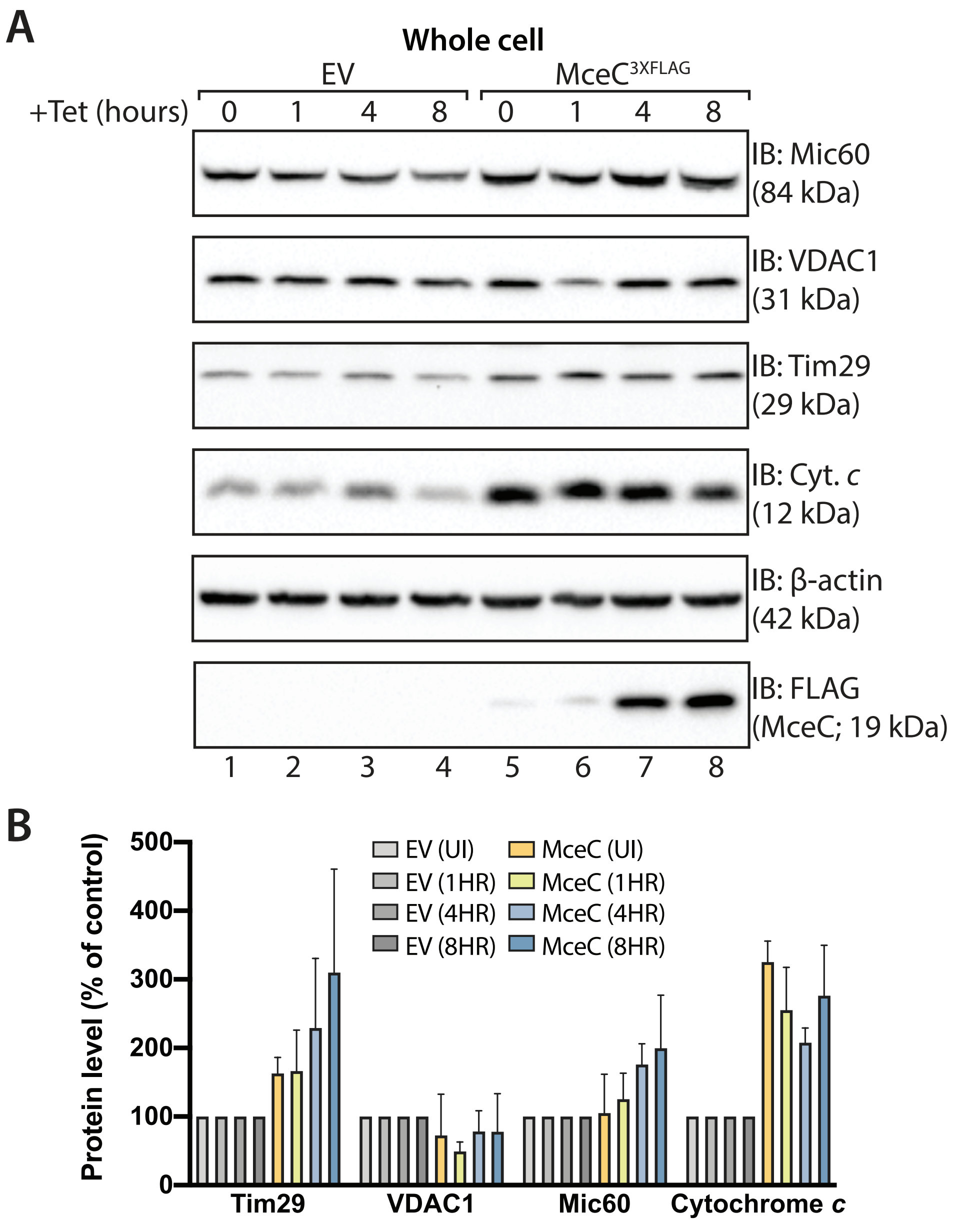

Fielden et al., 2020_Supplementary Figure 4
